# Effects of obstructive sleep apnea on circulating immune cells profiles: Evidence from NHANES dataset and Mendelian randomization

**DOI:** 10.1016/j.bjorl.2026.101810

**Published:** 2026-06-01

**Authors:** Yajie Jia, Xiang Gao, Shenglong Xu, Jingli Shi, Haifeng Hou, Yanru Li, Demin Han

**Affiliations:** a Capital Medical University, Beijing Tongren Hospital, Department of Otolaryngology Head and Neck Surgery, Beijing, China; b Key Laboratory of Otorhinolaryngology Head and Neck Surgery (Capital Medical University), Ministry of Education, Beijing, China; c Capital Medical University, Obstructive Sleep Apnea-Hypopnea Syndrome Clinical Diagnosis and Therapy and Research Centre, Beijing, China; d Edith Cowan University, School of Medical and Health Sciences, Centre for Precision Health, Perth, Australia; e Shandong First Medical University and Shandong Academy of Medical Sciences, School of Public Health, Taian, China; f Xinjiang Medical University, Xinjiang Key Laboratory of Biopharmaceuticals and Medical Devices, Xinjiang, China; g Department of Otorhinolaryngology Head and Neck Surgery, Peking university First Hospital, Beijing, China

**Keywords:** Obstructive sleep apnea, Circulating immune cells, National Health and Nutrition Examination Survey (NHANES), Mendelian randomization

## Abstract

**Objectives:**

This study aimed to investigate the impact of Obstructive Sleep Apnea (OSA) on immune cell profiles by utilizing both the National Health and Nutrition Examination Survey (NHANES) dataset and a two-sample Mendelian Randomization (MR) method.

**Methods:**

We included 20,732 participants from the NHANES to explore the relationship between symptom defined OSA and peripheral immune cells. Additionally, we conducted a two-sample MR analysis to investigate the effect of OSA on 35 summarized peripheral immune cell properties and 731 immunophenotypes. To ensure the robustness of findings in MR analysis, we conducted a sensitivity analysis.

**Results:**

The NHANES dataset and MR analysis indicated that OSA patients exhibit significantly elevated levels of white blood cells, neutrophils, and granulocytes, along with lower levels of lymphocytes. Furthermore, the MR analysis demonstrated a causal link between genetic predisposition to OSA and ten lymphocyte subtypes. Notably, there is a decrease in CD3 expression on Natural Killer-T (NKT) cells (*β*_IVW_ = −0.47, SE = 0.17, p = 0.005), and an increase in lgD expression on lgD + CD38+ B-cells (*β*_IVW_ = 0.36, SE = 0.15, p = 0.020) among individuals with a genetic predisposition to OSA. No heterogeneity or pleiotropy was identified.

**Conclusion:**

OSA is associated with elevated neutrophils, granulocytes, and systemic immune-inflammation index. Causally, OSA reduces NKT cells and disrupts lymphocyte homeostasis, impairing B-cell maturation/activation. These immune signatures may serve as clinical biomarkers for guide risk stratification and guide personalized therapies, including targeted immunomodulation.

**Level of evidence:**

This study combined a cross-sectional analysis of NHANES datasets with a Mendelian Randomization (MR) approach utilizing GWAS datasets. The MR analysis provides a level of evidence second only to randomized controlled trials, surpassing that of cohort and case-control studies.

## Introduction

Obstructive Sleep Apnea (OSA), characterized by breathing interruptions during sleep, impacts an estimated 936 million people globally.^1^ OSA results from the collapse of the upper airways, commonly leading to sleep fragmentation and Chronic Intermittent Hypoxia (CIH).^2^ The up-regulation of superoxide ions (oxygen radicals) induced by OSA exposes individuals to a pro-inflammatory condition, leading to immune dysfunction.^3^

Circulating blood cell phenotypes, vital indicators of immune system activity, are pivotal in the pathogenesis of diseases.^4^ Recent research has revealed that peripheral blood lymphocytes develop and differentiate into distinct subsets, playing crucial roles in regulating immune functions and the development of cancer and cardiovascular events.^5^^,^^6^ Previous studies have suggested a potential correlation between OSA and increased dysregulation in conventional blood cell counts, e.g., white blood cell, neutrophil, average platelet volume, Neutrophil-Lymphocyte Ratio (NLR), and Systemic Immune-Inflammation Index (SII).^7^, ^8^, ^9^ Moreover, studies have observed an association between OSA and CD3+, CD4+, and CD8+ T-lymphocyte profiles in peripheral blood.^10^^,^^11^ However, inconsistent findings exist due to limited sample size and clinical heterogeneity.^7^^,^^12^ Furthermore, there might be bidirectional links between sleep and the immune system,^13^ which bias the causal associations between circulating blood cell phenotypes and OSA.

Recent decades, NHANES employs a stratified, multistage probability sampling design to recruit approximately 5,000 U.S. participants annually, representing diverse, non-institutionalized populations across all age groups.^14^ This large, population-based cross-sectional sample enhances statistical power for detecting health outcome-risk factor associations. However, the cross-sectional study cannot identify the causality between exposure and outcomes. Impressively, recent Genome-Wide Association Studies (GWAS) have identified hundreds of Single Nucleotide Polymorphisms (SNPs) associated with OSA-related traits or circulating blood cell indices.^15^ The Mendelian Randomization (MR) approach leverages the random allocation and immutable nature of the human genome, allowing researchers to examine the causal relationship between exposure and a disease or medical condition.^16^ Thus, we conducted MR analysis to validate the findings in the cross-sectional study.

Building on the insights from previous studies, we employed a dual analytical framework to explore the potential relationship: Cross-sectional analysis of the NHANES dataset to identify OSA-immune associations while controlling for confounders; Two-sample MR with GWAS summary statistics to infer causality, leveraging the available genetic databases. This combined approach mitigates weaknesses inherent to observational studies (e.g., reverse causation) while compensating for MR’s reliance on genetic architecture assumptions.

## Methods

### Ethical approval

The studies involving human participants received approval from the NHANES Institutional Review Board and the Ethics Review Board of the National Center for Health Statistics. All study procedures adhered to the principles outlined in the Declaration of Helsinki. Informed written consent was obtained from all participants and, where applicable, from their legal guardians.

### Overall study design

**Fig. 1:**
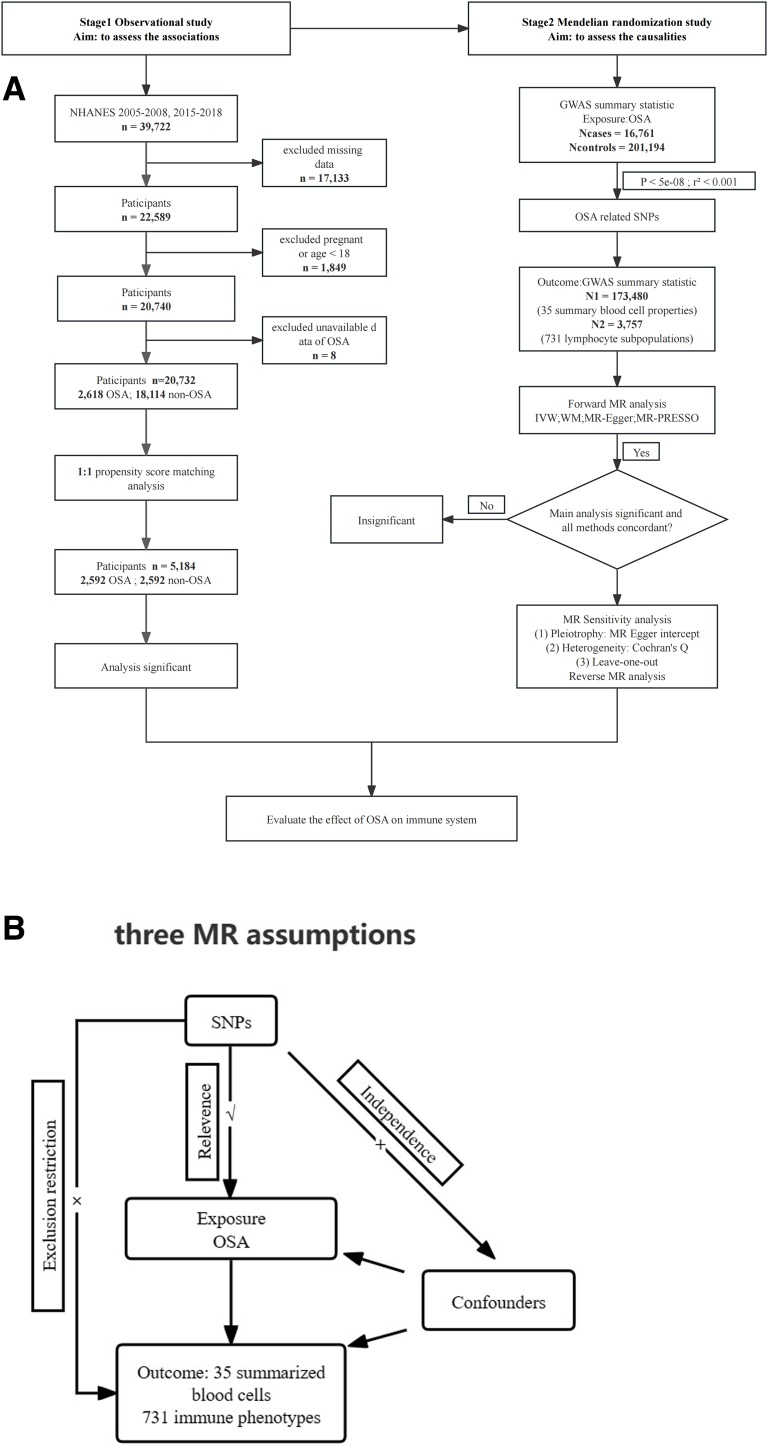
Study design. (A) Overall design of NHANES and Mendelian randomization analysis in the study. (B) The conceptual schematic of the MR research design.

This study was conducted in two stages (Fig. 1A). First, we performed a cross-sectional analysis of the NHANES database using Propensity Score Matching (PSM), during which we carried out a logistic regression analysis to determine the Odds Ratio (OR) and its Confidence Interval (CI) between OSA and peripheral immune cells. Since the cross-sectional study cannot identify the causality between exposure and outcomes, we conducted MR analysis to support our findings in stage 1. The conceptual schematic of the MR research design is illustrated in Fig. 1B: 1) Genetic variants must be strongly linked with exposure; 2) They should be free from potential confounding factors; 3) These genetic instruments should influence the outcome solely through exposure. We reported our findings following the guidelines outlined in the Strengthening the Reporting of Observational Studies in Epidemiology (STROBE) reporting guideline (Supplementary Table S1).^17^

### Data sources and study population

Every 2-years, the NHANES program collects nationally representative data from approximately 10,000 randomly selected participants. These individuals complete comprehensive health interviews covering demographic, socioeconomic, dietary, and health-related factors, followed by physical examinations and laboratory testing.^14^ The 2005–2008 and 2015–2018 NHANES datasets were selected because they are the only survey periods that simultaneously included both questionnaire-based assessments (related to OSA) and laboratory-based peripheral blood cell examinations. A total of 17,133 individuals were excluded due to missing data from the Sleep Disorders Questionnaire or Complete Blood Count, eight were excluded due to missing OSA data, and 1,849 due to being pregnant or being under 18-years of age. Finally, 20,732 participants were included in the stage 1 analysis.

## Measures

### Outcomes: peripheral immune cells

The clinical hematological parameters examined in this study included Complete Blood Count, including White Blood Cell (WBC), Lymphocyte (Lym), Monocyte (Mono), segmented Neutrophils (Nsg), Eosinophils (Eos), Basophils (Baso), Red Blood Cell (RBC), Hemoglobin (HGB), Hematocrit (HCT), Mean Cell Volume (MCV), Mean Cell Hemoglobin (MCH), Mean Cell Hemoglobin Concentration (MCHC), Red Cell Distribution Width (RDW), Platelet Count (PLT), and Mean Platelet Volume (MPV). In addition, participants' inflammatory markers included NLR and SII, with NLR calculated as the neutrophil/lymphocyte count and SII calculated as platelet count multiplied by the neutrophil/lymphocyte count.^18^

### Exposure: OSA

The diagnosis was derived from three key criteria in the NHANES Sleep Disorders Questionnaire: (1) Snoring ≥3 nights/week, (2) Nocturnal apneas ≥3 nights/week, and 3) Excessive daytime sleepiness (≥1 episode/month despite ≥7 h nightly sleep). All three criteria were required for classification.^19^

### Covariates

The covariates were selected based on their established roles in OSA pathogenesis and their potential to confound immune-inflammatory outcomes.^20^^,^^21^ Finally, the Covariates adjusted for in this study included age, sex, race, Body Mass Index (BMI), and diagnoses of hypertension, high cholesterol, and diabetes. Diagnoses were defined as participants ever being told by a doctor that they had these conditions.^22^ These factors influence blood cell parameters through multiple pathways, including age-related immunosenescence, sex hormone-mediated immune regulation, racial differences in baseline profiles, obesity-associated inflammation, and metabolic disorder-induced systemic inflammation.^23^^,^^24^

### Statistical analysis

Independent samples *t*-tests for continuous variables and Chi-Squared tests for categorical variables were used to compare differences in baseline characteristics between the OSA and non-OSA groups. Multiple imputation was employed to handle missing values, using model estimation and repeated simulations were used to generate a complete set of datasets.^25^

To analyze the relationship between OSA and peripheral immune cells, the PSM method, based on logistic regression models,^26^ was applied to adjust for selection bias and potential confounders. To mitigate potential selection bias in patient allocation, we performed Propensity Score Matching (PSM) between the OSA and non-OSA groups using a 1:1 matching protocol with a caliper width of 0.02. This caliper setting was empirically optimized from the theoretical threshold (0.2×σ_logit = 0.116) to achieve optimal covariate balance, as suggested by established diagnostic methods.^27^ All data in this study were statistically analyzed using *R* (version 4.3.2).

### Mendelian randomization

#### Data sources

The OSA summary-level data were obtained from a recently published GWAS, which included 16,761 OSA patients and 201,194 controls in the FinnGen study. The characteristics of the genetic data sources used are summarized in Supplemental Table S2.^28^ OSA was diagnosed using the International Classification of Diseases, 10th edition (ICD-10) and 9th edition (ICD-9) codes (ICD-10: G47.3, ICD-9: 3472A), based on subjective symptoms, clinical examination, and sleep registration using the apnea-hypopnea index of five per hour or respiratory event index of five per hour. Principal covariates, such as age and sex, were adjusted in the association tests for all sources. Astle et al. performed GWAS on the blood cell properties of 173,480 participants of European-ancestry and reported 35 summary blood cell properties, which could be obtained from the IEU OpenGWAS Database (https://gwas.mrcieu.ac.uk). There were 731 immunophenotypes for cellular subpopulation analyses, with 3,757 individuals profiled by flow cytometry using the Sardinian founder population.^29^ More information on the GWAS datasets for OSA and circulating immune cell phenotypes is shown in Table S2. We examined SNPs associated with confounders or outcomes using LDlink (Https://ldlink.nih.gov/), an alternative method of the Phenoscanner database. SNPs linked to previously identified confounding factors related to circulating immune cells, such as blood pressure and diabetes,^24^ were not found in LDlink’s analysis. However, SNPs associated with OSA were related to BMI phenotypes. Further details on IVs associated with confounders in reverse MR analyses can be found in Supplementary Table S3.

#### MR analysis

The study was carried out using the TwoSampleMR (version 0.5.7) software package based on the *R* platform (version 4.3.2). We extracted genome-wide Single Nuclear Polymorphisms (SNPs) for OSA and hematological traits with a p < 5 × 10^−8^ criterion.^30^ Since the p < 5 × 10^−8^ threshold could detect only a few Instrumental Variables (IVs) for several hematological traits, we chose SNPs with p < 5 × 10^−6^ as the reverse IVs criterion.^31^ This adjustment enabled robust Mendelian randomization analyses, particularly for the Inverse-Variance Weighted (IVW) method – the gold-standard MR approach that achieves optimal statistical power when adequate valid IVs are available.^17^ To ensure that SNPs’ effects on OSA and hematological traits were related to the same allele, we aligned the direction of effects across all SNPs. Additionally, with a window of 10,000 kb, we eliminated SNPs that were in chained disequilibrium (*r*^2^ < 0.001) and a Minimum Allele Frequency (MAF) of 0.01, and retrieved the remaining SNPs. To ensure robust exposure, we calculated the *F*-statistic and concluded that an *F*-statistic of 10 would be sufficient to counteract weak instrumental bias,^32^ as shown in Supplementary Table S4‒S5. To obtain reliable causal effects, we used Inverse-Variance Weighted (IVW) methods as the principal analytical strategy.^33^ More details on MR sensitivity analysis and reverse MR analysis can be found in the previous study.^30^

## Results

### Population characteristics of NHANES

A total of 20,732 participants (10,277 men and 10,455 women) were included in the final analysis. Of these, 2,618 participants (12.63%) met the criteria for probable OSA based on self-reported symptoms (snoring, nocturnal apneas, and daytime sleepiness). Compared to non-OSA participants, those with OSA were more likely to be older white men, have a higher BMI, and suffer from hypertension, diabetes, and higher cholesterol levels (p < 0.001).

### Association between OSA and hematological parameters

After 1:1 propensity score matching, 2,592 subjects were included in both the OSA and non-OSA groups for the analyses. There were no significant differences between the two groups in terms of age, sex, race, BMI, diagnosis of hypertension, high cholesterol, and diabetes. Before matching, participants with OSA had significantly higher levels of white blood cell, monocyte, segmented neutrophils, eosinophils, basophils, NLR, SII (all p < 0.001), hemoglobin, hematocrit, red blood cell and red cell distribution width (p < 0.05). After adjusting for confounding factors, significant differences in the prevalence of OSA were found in these indicators: white blood cells, monocytes, segmented neutrophils, basophils, mean cell volume, and platelet count (all p < 0.05). In addition, participants with OSA had significantly higher levels of SII (p < 0.05), with a trend towards significance in the NLR (p = 0.091).

### Cross-sectional sensitivity analyses

**Fig. 2:**
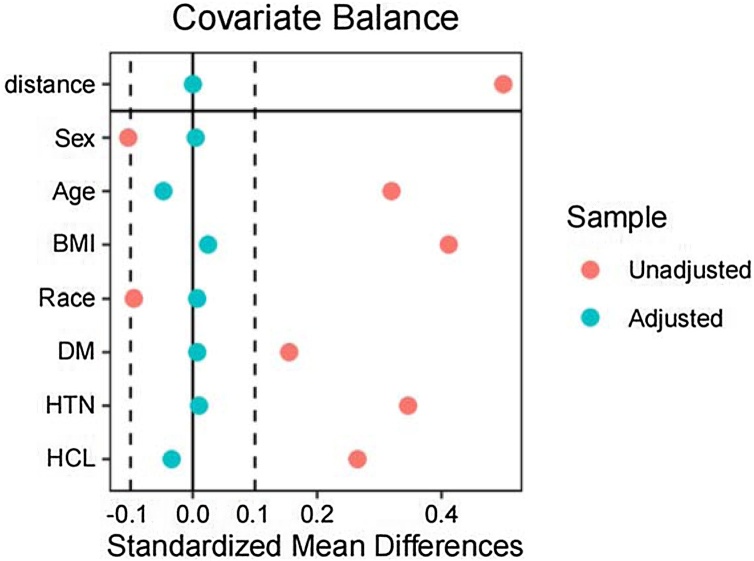
Covariate balance before and after propensity score matching. BMI, Body Mass Index; DM, Diabetes; HCL, High Cholesterol; HTN, Hypertension.

In the cross-sectional section of the study, post-matching Standardized Mean Differences (SMDs) were all < 0.1, confirming successful balancing, while retaining 99% of the OSA cohort for robust statistical power. Covariate balance before and after propensity score matching was visualized in Fig. 2.

In addition, we employed multiple imputation to handle missing data (e.g., BMI, hypertension, hyperlipidemia) and subsequently performed propensity score matching, resulting in 2,592 well-balanced matched pairs. Complete-case analysis within matched pairs (n = 2,205 non-OSA; n = 2,233 OSA) consistently replicated originally significant hematological findings (WBC, monocytes, neutrophils, MCV, platelets, SII; all p < 0.05), with only MCH showing borderline significance (p = 0.055). Crucially, the minimal discrepancy between approaches (Supplementary Table S6) confirms this section results regarding OSA-immune associations.

### MR of OSA and circulating immune cells

**Fig. 3:**
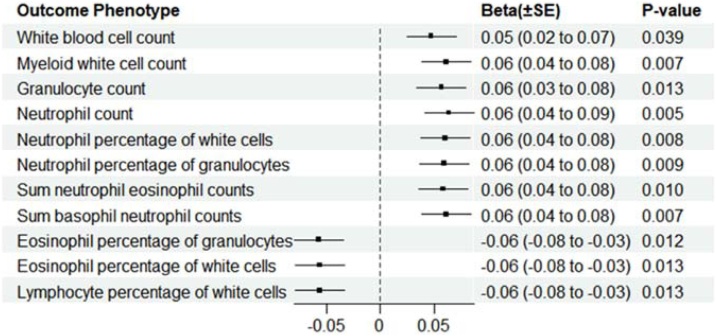
Forest plot of MR showing estimates of genetically predicted OSA on Peripheral circulating immune cells.

The casual effects of genetically predicted OSA on peripheral circulating immune cells were shown in Fig. 3. In general, consistent with the observational study, genetic liability to OSA was associated with an increased count of white blood cells (*β*_IVW_ = 0.05, SE = 0.02, p = 0.038) and myeloid white cells (*β*_IVW_ = 0.06, SE = 0.02, p = 0.007). Importantly, OSA also increased the count of neutrophils (*β*_IVW_ = 0.06, SE = 0.02, p = 0.005) and granulocytes (*β*_IVW_ = 0.06, SE = 0.02, p = 0.010). Moreover, there was evidence for a positive effect of OSA on the sum counts of neutrophil and eosinophils (*β*_IVW_ = 0.06, SE = 0.02, p = 0.010) and basophil and neutrophils (*β*_IVW_ = 0.06, SE = 0.02, p = 0.007), as well as the neutrophil percentage of white cells (*β*_IVW_ = 0.06, SE = 0.02, p = 0.008) and the neutrophil percentage of granulocytes (*β*_IVW_ = 0.06, SE = 0.02, p = 0.009).

In contrast, although the sum counts of neutrophils and eosinophils were increased, genetically liability to OSA was associated with a decreased eosinophil percentage of white cells (*β*_IVW_ = −0.06, SE = 0.02, p = 0.013) and eosinophil percentage of granulocytes (*β*_IVW_ = −0.06, SE = 0.02, p = 0.012). Interestingly, we found that OSA decreased the lymphocyte percentage of white cells (*β*_IVW_ = −0.06, SE = 0.02, p = 0.012), which is inconsistent with the cross-sectional study. Thus, we extended our analyses by further measuring the causal estimates of OSA on lymphocyte cellular subpopulations.

**Table 1:** Characteristics of participants with OSA before and after PSM Analysis in the NHANES 2005‒2008 and 2015–2018.

Characteristics	Before Matching				After Matching			
	Total	non-OSA	OSA	p	Total	non-OSA	OSA	p
N	20732	18114	2618		5184	2592	2592	
Age (y), Mean (SD)	48.7 (18.9)	48.0 (19.1)	53.4 (16.7)	<0.001	53.7 (17.4)	54.1 (18.0)	53.3 (16.7)	0.102
Sex (%)				<0.001				0.889
Female	10455 (50.4%)	9253 (51.1%)	1202 (45.9%)		2370 (45.7%)	1182 (45.6%)	1188 (45.8%)	
Male	10277 (49.6%)	8861 (48.9%)	1416 (54.1%)		2814 (54.3%)	1410 (54.4%)	1404 (54.2%)	
Race (%)				<0.001				0.081
Non-Hispanic White	8405 (40.5%)	7242 (40.0%)	1163 (44.4%)		2257 (43.5%)	1107 (42.7%)	1150 (44.4%)	
Non-Hispanic Black	4486 (21.6%)	3920 (21.6%)	566 (21.6%)		1174 (22.6%)	620 (23.9%)	554 (21.4%)	
Mexican American	3641 (17.6%)	3235 (17.9%)	406 (15.5%)		830 (16.0%)	424 (16.4%)	406 (15.7%)	
Others	4200 (20.3%)	3717 (20.5%)	483 (18.4%)		923 (17.8%)	441 (17.0%)	482 (18.6%)	
BMI (kg/m²)	29.1 (7.01)	28.7 (6.78)	32.0 (7.92)	<0.001	31.6 (7.49)	31.5 (7.47)	31.7 (7.52)	0.351
Diabetes (%)				<0.001				0.347
No	17579 (86.7%)	15557 (87.7%)	2022 (79.6%)		4049 (80.5%)	2037 (81.1%)	2012 (80.0%)	
yes	2695 (13.3%)	2177 (12.3%)	518 (20.4%)		978 (19.5%)	475 (18.9%)	503 (20.0%)	
Hypertension (%)				<0.001				0.739
No	13573 (65.5%)	12255 (67.7%)	1318 (50.3%)		2647 (51.1%)	1330 (51.3%)	1317 (50.8%)	
yes	7159 (34.5%)	5859 (32.3%)	1300 (49.7%)		2537 (48.9%)	1262 (48.7%)	1275 (49.2%)	
High cholesterol level (%)				<0.001				0.232
No	13394 (64.6%)	12005 (66.3%)	1389 (53.1%)		2720 (52.5%)	1338 (51.6%)	1382 (53.3%)	
Yes	7338 (35.4%)	6109 (33.7%)	1229 (46.9%)		2464 (47.5%)	1254 (48.4%)	1210 (46.7%)	
WBC (10^9^/L)	7.31 (3.69)	7.24 (2.49)	7.79 (8.06)	<0.001	7.58 (6.12)	7.38 (3.08)	7.78 (8.09)	0.020
Lym (10^9^/L)	2.23 (2.88)	2.21 (1.49)	2.39 (7.08)	0.194	2.30 (5.29)	2.23 (2.35)	2.38 (7.11)	0.306
Mono (10^9^/L)	0.57 (0.20)	0.57 (0.20)	0.60 (0.21)	<0.001	0.59 (0.21)	0.58 (0.20)	0.60 (0.21)	0.030
Nsg (10^9/L)	4.26 (1.69)	4.22 (1.66)	4.54 (1.86)	<0.001	4.42 (1.76)	4.31 (1.64)	4.53 (1.87)	<0.001
Eos (10^9^/L)	0.21 (0.17)	0.20 (0.17)	0.22 (0.18)	<0.001	0.22 (0.17)	0.22 (0.17)	0.22 (0.18)	0.701
Baso (10^9^/L)	0.05 (0.06)	0.05 (0.06)	0.06 (0.07)	<0.001	0.05 (0.06)	0.05 (0.06)	0.06 (0.07)	0.001
RBC (10^12^/L)	4.73 (0.51)	4.73 (0.50)	4.75 (0.53)	0.025	4.76 (0.52)	4.76 (0.51)	4.75 (0.53)	0.709
HGB (g/dL)	14.1 (1.56)	14.1 (1.56)	14.2 (1.59)	0.004	14.2 (1.59)	14.2 (1.58)	14.2 (1.59)	0.246
HCT (%)	41.9 (4.30)	41.8 (4.29)	42.1 (4.37)	0.001	42.1 (4.33)	42.0 (4.28)	42.2 (4.38)	0.303
MCV (fL)	88.7 (5.94)	88.7 (5.93)	88.9 (6.02)	0.057	88.8 (6.08)	88.6 (6.13)	89.0 (6.02)	0.029
MCH (pg)	29.9 (2.43)	29.9 (2.43)	30.0 (2.44)	0.300	29.9 (2.50)	29.9 (2.56)	30.0 (2.43)	0.055
MCHC (g/dL)	33.7 (1.05)	33.7 (1.05)	33.7 (1.05)	0.211	33.7 (1.05)	33.7 (1.06)	33.7 (1.05)	0.447
RDW (%)	13.4 (1.37)	13.3 (1.37)	13.5 (1.38)	<0.001	13.5 (1.41)	13.6 (1.46)	13.5 (1.36)	0.211
PLT (10^9^/L)	256 (68.3)	256 (68.0)	256 (70.1)	0.930	254 (70.1)	252 (70.1)	256 (70.1)	0.034
MPV (fL)	8.13 (0.91)	8.13 (0.91)	8.16 (0.91)	0.129	8.16 (0.91)	8.16 (0.90)	8.16 (0.91)	0.940
NLR (%)	2.13 (1.16)	2.12 (1.16)	2.23 (1.16)	<0.001	2.20 (1.14)	2.18 (1.12)	2.23 (1.16)	0.091
SII	548 (346)	545 (346)	573 (346)	<0.001	560 (339)	548 (332)	572 (345)	0.013

All the quantitative data were expressed with mean and Standard Deviation (SD); BMI, Body Mass Index; WBC, White Blood Cell; Lym, Lymphocyte; Mono, Monocyte; Nsg, Segmented Neutrophils; Eos, Eosinophils; Baso, Basophils; RBC, Red Blood Cell; HGB, Hemoglobin; HCT, Hematocrit; MCV, Mean Cell Volume; MCH, Mean Cell Hemoglobin; MCHC, Mean Cell Hemoglobin Concentration; RDW, Red cell Distribution Width; PLT, Platelet count; MPV, Mean Platelet Volume; NLR, Neutrophil-To-Lymphocyte Ratio; SII, Systemic Immune Inflammation Index.

**Fig. 4:**
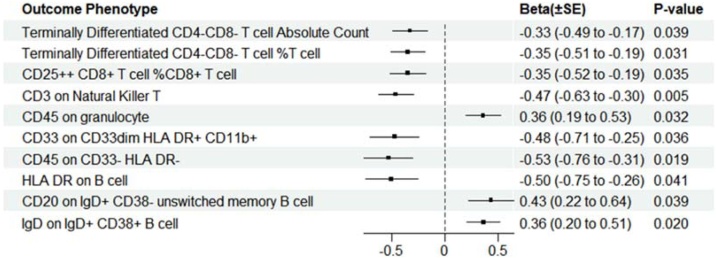
Forest plot of MR showing estimates of genetically predicted OSA on circulating lymphocyte subpopulations.

Regarding T-cells, the results indicated that OSA was associated with decreased counts of terminally differentiated CD4-CD8- T cells (*β*_IVW_ = −0.33, SE = 0.16, p = 0.039), the percentage of CD25++ CD8+ T-cells on CD8+ T-cells (*β*_IVW_ = −0.35, SE = 0.16, p = 0.034) and CD3 on Natural Killer T-cells (*β*_IVW_ = −0.47, SE = 0.17, p = 0.005). In the analyses of B-cells, there was evidence that genetically determined OSA may decrease the HLA-DR on B cells (*β*_IVW_ = −0.50, SE = 0.25, p = 0.041). In contrast, there were evidence that the number of CD20 on IgD + CD38- unswitched memory B-cells (*β*_IVW_ = 0.43, SE = 0.21, p = 0.039) and IgD on IgD + CD38+ B-cells (*β*_IVW_ = 0.36, SE = 0.15, p = 0.020) was increased. More details on the subsets analysis are provided in Fig. 4 and Supplemental Table S7.

### Sensitivity analysis and reverse MR analysis

There was no evidence of potential heterogeneity or horizontal pleiotropy in the genetic prediction of OSA and outcome, with comprehensive results detailed in Supplementary Table S3. The visualization of results, including scatter plot, forest plot, funnel plot, and leave-one-out analyses is shown in Figures S1–S23. The reverse MR analysis revealed that all positive results were not biased by bi-directional effects (see Supplementary Table S8).

## Discussion

To the best of our knowledge, this study is the first to use cross-sectional NHANES data and two-sample MR with a large genetic database to identify the effects of obstructive sleep apnea on circulating immune cells. Not only the NHANES analyses linked symptom based OSA risk (characterized by self-reported snoring, nocturnal apnea, and daytime sleepiness) to elevated inflammatory markers (e.g., white blood cell, lymphocyte, mean platelet volume), which has been reported in previous meta-analyses, but also have established associations between OSA and novel hematological indices (NLR and SII). Impressively, our study advances the field by providing the first causal evidence, via GWAS-based Mendelian randomization, linking OSA to specific leukocyte alterations (myeloid cells, neutrophils, granulocytes, eosinophils, and lymphocytes). Beyond confirming prior observations, we found that OSA causally disrupts lymphocyte homeostasis – particularly in key immunosurveillance populations such as NKT cells and terminally differentiated CD4-CD8- T-cells. These findings suggest that OSA-associated immune dysfunction may mechanistically contribute to its comorbidities, positioning these immune subsets as potential biomarkers for risk stratification and therapeutic targets (e.g., immunomodulation in OSA-related cancers).

Our findings demonstrated that OSA was causally associated with an increased counts of white blood cells, granulocytes, and neutrophil counts, as estimated by both cross-sectional and MR results, which is consistent with previous observational findings.^7^ Several mechanisms, including chronic hypoxia, sympathetic overactivity, oxidative stress, and endothelial dysfunction, have been implicated in the relationship between OSA and changes in circulating blood cells.^34^ Regarding the decrease in lymphocytes observed in the MR findings, some researchers have shown that lower lymphocyte counts are associated with hypothalamic-pituitary-adrenal axis activation, increased systemic cortisol production, and altered sleep patterns.^35^ In addition, our NHANES results after propensity score matching analyses showed that OSA was associated with a higher level of MCV and PLT, which have been less reported in previous studies. Nena et al. suggested that hypoxia may have an activating effect on platelet function, specifically, thrombocytes have Cyclooxygenase (COX) enzymes that can stimulate the synthesis of prostanoids, such as the inflammatory thromboxane A2, making them a potential source of inflammation.^36^ Last but not least, SII may be a more comprehensive representation of the balanced state of immune and inflammatory conditions,^37^ and has been recognized as a strong predictor of mortality from cardiovascular diseases.^38^ The validation of elevated SII levels as indicators of heightened inflammation in OSA was further confirmed in our large population-based study.

Our findings indicate decreased CD3 expression on NKT cells in individuals with OSA, consistent with the research conducted by Gaoatawe et al.^39^ Their study revealed that patients with severe OSA exhibited an inverse correlation between the number of invariant NKT cells and factors such as apnea-hypopnea index and SpO_2_% <90%. A reduced count of NKT cells has been associated with increased vulnerability to atherosclerotic plaque instability and cancer immunity.^39^^,^^40^ T-cells and B-cells are crucial in regulating immune responses and inflammatory processes, however, their relationship to OSA remains relatively poorly understood. Our MR analysis revealed that genetically predicted OSA causally increase in Immunoglobulin D (IgD) levels on IgD + CD38+ B-cells and CD20 on IgD + CD38- unswitched memory B-cells. IgD serves as a marker of B-cell maturation, and unswitched memory B-cells are usually at an earlier stage of B-cell development. Our findings suggest that OSA promotes the maturation process of B-cells, indicating a body’s stress-induced pro-inflammatory response. This observation aligns with similar studies that have demonstrated an increase in the expression of CD19+, a cell surface marker typically associated with the development and activation of B-cells.^41^ In addition to promoting the maturation of B-cells, the findings also indicate that the reduction in terminally differentiated CD4-CD8- T-cells and the dual pattern of CD45 expression (a decrease in CD45 on CD33- HLA-DR- and an increase in CD45 on granulocytes) suggest a dynamic shift towards activated immune cells in the immune cell composition, potentially signifying alterations in the immune response.^10^

From the perspective of pathophysiological mechanisms, emerging evidence suggests that OSA-related pathophysiological mechanisms-including CIH, sleep fragmentation, and sympathetic overactivation-disrupt neuroendocrine-immune homeostasis, leading to impaired innate and adaptive immunity. These alterations may foster a permissive microenvironment for tumorigenesis, accelerate cancer progression, and contribute to therapy resistance in malignancies such as lung cancer.^42^ Go deeper, a key mechanistic link involves TGF-β, a pleiotropic cytokine with dual roles in tumor suppression and promotion. In the tumor microenvironment, TGF-β signaling modulates immune evasion by suppressing cytotoxic T-cell and NK-cell activity (which is consist with our study results) while promoting the expansion of immunosuppressive cell populations (e.g., Tregs, MDSCs).^43^ In addition, OSA-associated HIF-1α, overexpressed in many cancers, not only drives treatment resistance but also upregulates TGF-β in monocytes and NK cells, inducing an immunosuppressive phenotype.^44^ From Clinical evidences, monocytes from untreated OSA patients exhibit elevated TGF-β secretion, which not only suppresses Natural Killer (NK) cell cytotoxicity but also enhances lung cancer cell migration ‒ a defect reversible upon CPAP-mediated reoxygenation.^45^

From the perspective of clinical standpoint, our findings highlight that OSA-associated immune dysregulation ‒ particularly neutrophil hyperactivation and NKT cell depletion ‒ may mechanistically contribute to comorbidities such as cardiovascular disease and cancer.^38^^,^^39^ Clinically, these immune alterations could serve as biomarkers for risk stratification (e.g., identifying patients with heightened inflammatory or oncogenic potential) and have potential to guide personalized management strategies, including targeted immunomodulation (e.g., neutrophil-inhibiting therapies or NKT cell reconstitution) alongside conventional OSA therapies.

Moving forward, with the advent of artificial intelligence and advanced portable monitoring technologies, farther validation of our findings using objective OSA diagnostic tools (e.g., home sleep apnea testing or polysomnography) in large-scale population studies would be of significant interest. Additionally, well-designed randomized controlled trials should be conducted to systematically evaluate the differential effects of OSA therapies ‒ such as CPAP, surgical interventions, and alternative treatments ‒ on immune modulation, particularly T-cell and B-cell functional dynamics.

The strengths of our study include the use of a large, nationally representative cohort in combination with two-sample MR analyses to investigate the effect of OSA on circulating blood cell phenotypes. MR analyses are less susceptible to bias from reverse causation and confounding than traditional observational studies because they use genetic information as an instrumental variable. Additionally, propensity score matching analyses allowed for the simultaneous inclusion of multiple factors as covariates supported by the large sample size of NHANES. Thus, the consistency between the MR analyses and the observational study added robustness to the findings.

However, this study had several limitations. Firstly, although the cross-sectional findings could be influenced by recall bias due to NHANES’s self-reported symptoms, the combination of key symptoms (snoring, nocturnal apneas, and daytime sleepiness) may help attenuate potential confounding to some extent. Secondly, all GWAS participants in the MR study were of European origin which may limit the generalizability to other populations. Despite the ethnic differences, our results found overlapping and consistent results in NHANES and MR, reinforcing the cross-ethnic impact of OSA on the immunity system. Thirdly, a well-recognized challenge of PSM, particularly with 1:1 matching without replacement, often reduces sample size and statistical power due to unmatched cases. However, our study achieved 99% matching success for OSA cases, maintaining robust statistical power despite using stringent calipers. Although we adjusted for multiple relevant confounders, residual confounding from unmeasured factors may still exist. However, our Mendelian randomization analysis provided evidence supporting causal associations for some key findings. Fourthly, MR approaches for estimating causal effects using instrumental variables assume the exposure-outcome relationship is linear, hence *U*- or *J*-shaped effects may be overlooked. Future investigations are needed to identify the non-linear causality between sleep apnea measures and immune cell function. Finally, given the recognized heterogeneity of OSA phenotypes, our MR analysis could not account for potential differences related to daytime sleepiness. Future studies with more detailed phenotypic data are needed to identify the linear and the non-linear causality between sleep apnea measures and immune cell function.

## Conclusions

Our findings demonstrated that OSA is characterized by elevated levels of specific peripheral immune cells and SII, a newly established immune-inflammatory index. Importantly, our findings highlight that OSA-associated immune dysregulation ‒ particularly neutrophil hyperactivation and NKT cell depletion ‒ may mechanistically contribute to comorbidities such as cardiovascular disease and cancer. Clinically, these immune alterations could serve as biomarkers for risk stratification (e.g., identifying patients with heightened inflammatory or oncogenic potential) and guide personalized management strategies, including targeted immunomodulation (e.g., neutrophil-inhibiting therapies or NKT cell reconstitution) alongside conventional OSA therapies.

## ORCID ID

Yajie Jia: 0000-0002-5021-5304

Xiang Gao: 0000-0003-4095-3159

Shenglong Xu: 0000-0002-0607-4524

Jingli Shi: 0009-0007-3568-240X

Haifeng Hou: 0000-0002-1131-1619

Yanru Li: 0000-0002-6474-7141

Demin Han: 0000-0002-4379-407X

## Authors’ contributions

Yajie Jia and Xiang Gao performed data analysis and wrote the manuscript. Yajie Jia participated in the visualization of the results. Xiang Gao contributed to the verification of the underlying data. Shenglong Xu and Jingli Shi were in charge of the proof-reading and refinement of the manuscript. Haifeng Hou contributed to refining the manuscript. Demin Han and Yanru Li were involved in the concept and design of the study. All authors reviewed the content of the manuscript and approved the final version.

## Funding

This project was supported by Beijing Natural Science Foundation (L243032), 10.13039/501100001809National Natural Science Foundation of China (81970866), and Innovative Health Promotion Strategies for China's New Era (2019-XZ-29).

## Data availability statement

Nhanes data are available at https://www.cdc.gov/nchs/nhanes. The datasets analyzed in this Mendelian randomization study are publicly accessible summary statistics, and all summary data can be easily downloaded from the designated website: the IEU Open GWAS project https://gwas.mrcieu.ac.uk/ and https://www.finngen.fi/en.

## Declaration of competing interest

The authors declare no conflicts of interest.
