# Supplementary material for: Effects of obstructive sleep apnea on circulating immune cells profiles: Evidence from NHANES dataset and Mendelian randomization
Source: Braz J Otorhinolaryngol. 2026 Jun 1;92(4):101810. doi: 10.1016/j.bjorl.2026.101810 (PMC13241890; doi:10.1016/j.bjorl.2026.101810)
Supplement: Supplementary file 1 [file mmc1.pdf]

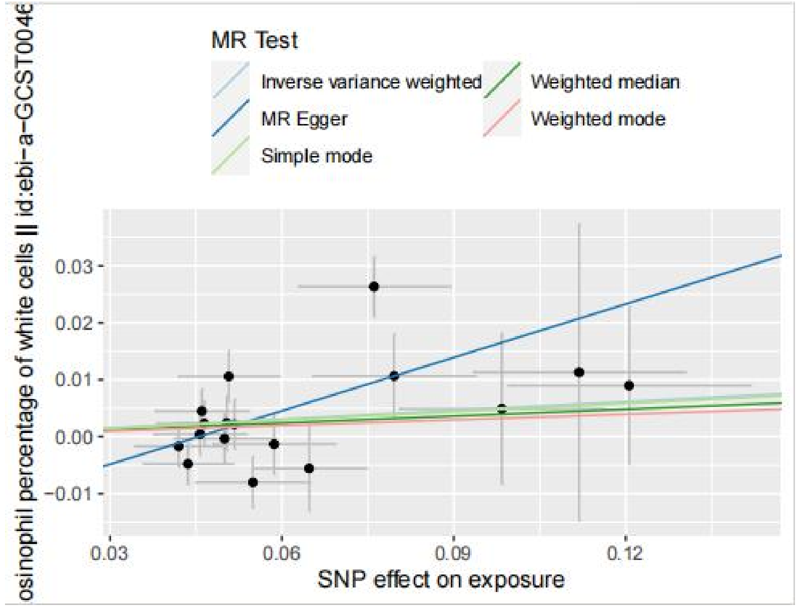

Scatter plot

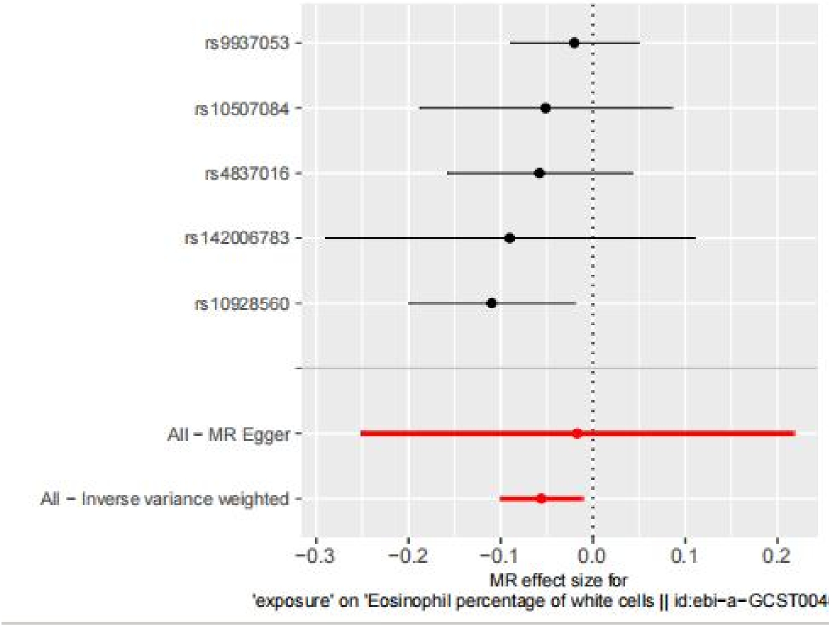

Forest plot

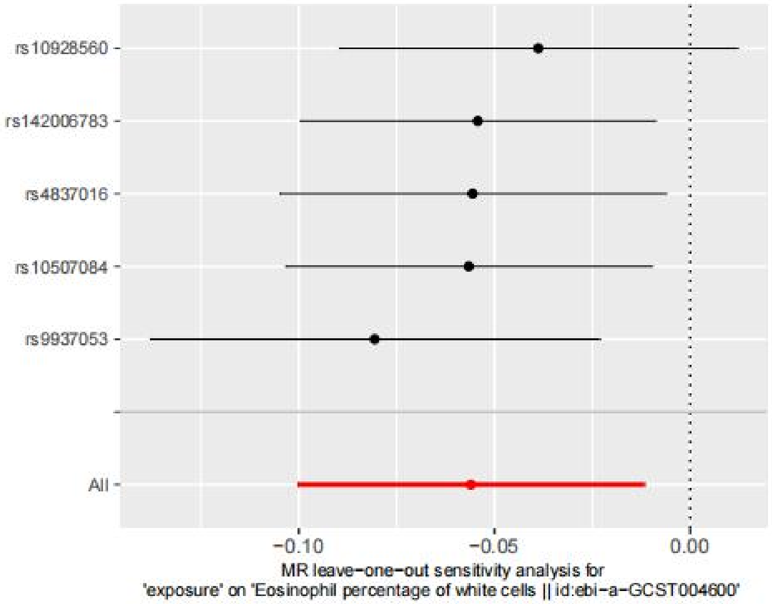

Leave-one-out analyses

Figure S1. Genetic predicted OSA was causally associated with decreased eosinophil percentage of white cells.

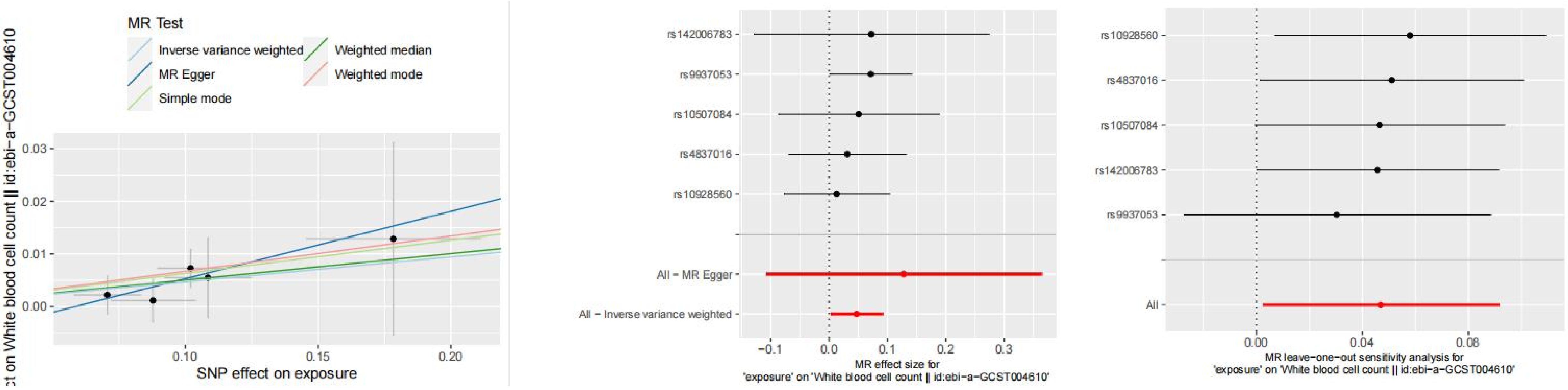

Scatter plot Forest plot Leave-one-out analyses

Figure S2. Genetic predicted OSA was causally associated with increased white blood cell count.

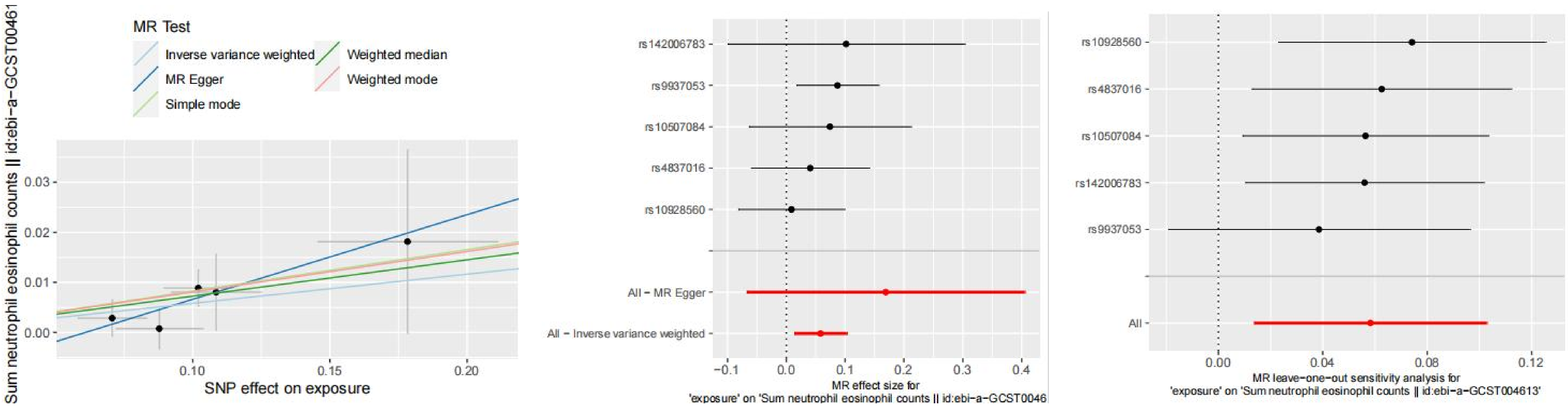

Scatter plot

Forest plot

Leave-one-out analyses

Figure S3. Genetic predicted OSA was causally associated with increased sum counts of neutrophil and eosinophil.

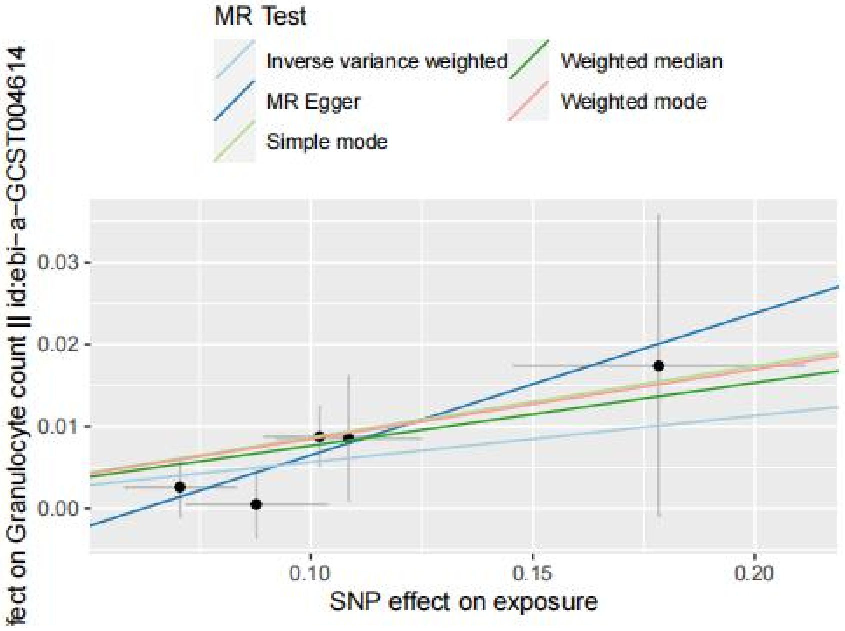

Scatter plot

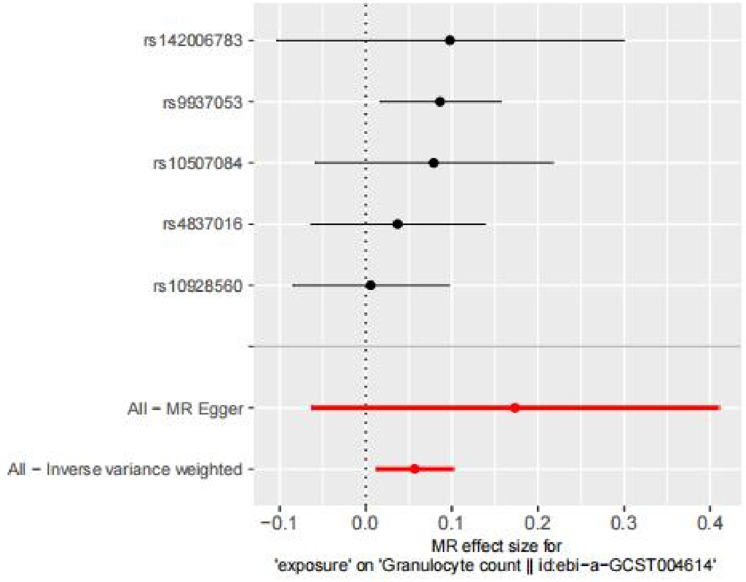

Forest plot

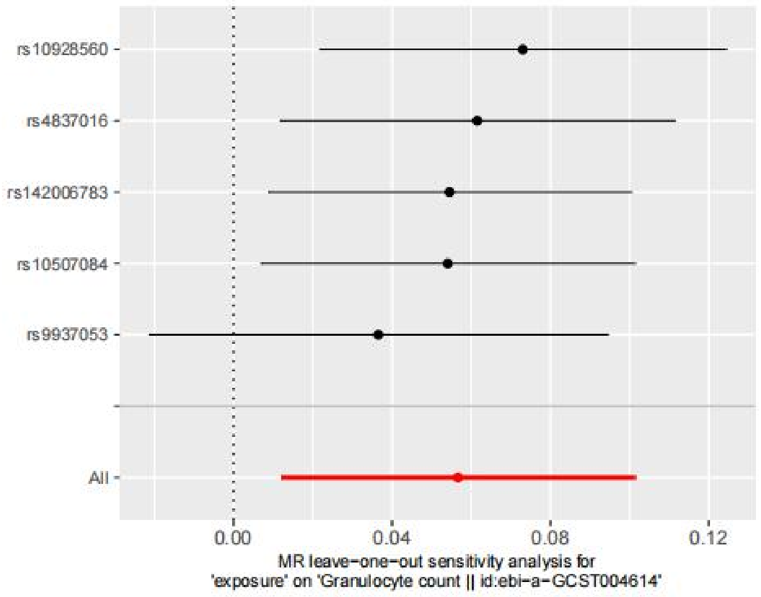

Leave-one-out analysis

Figure S4. Genetic predicted OSA was causally associated with increased granulocyte count.

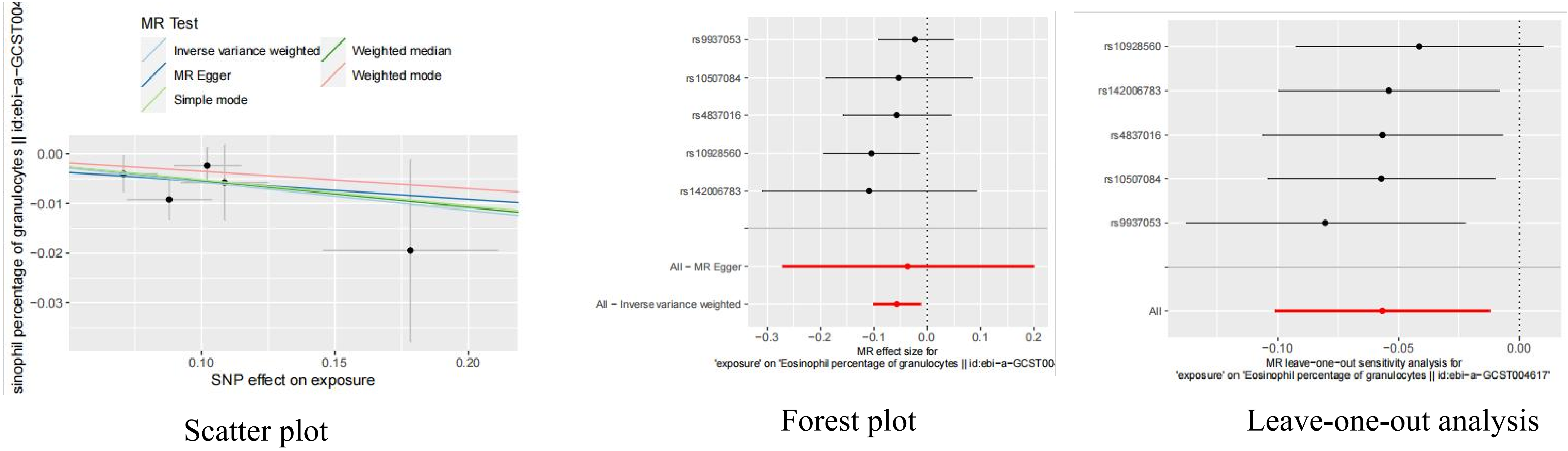

Figure S5. Genetic predicted OSA was causally associated with decreased eosinophil percentage on granulocytes.

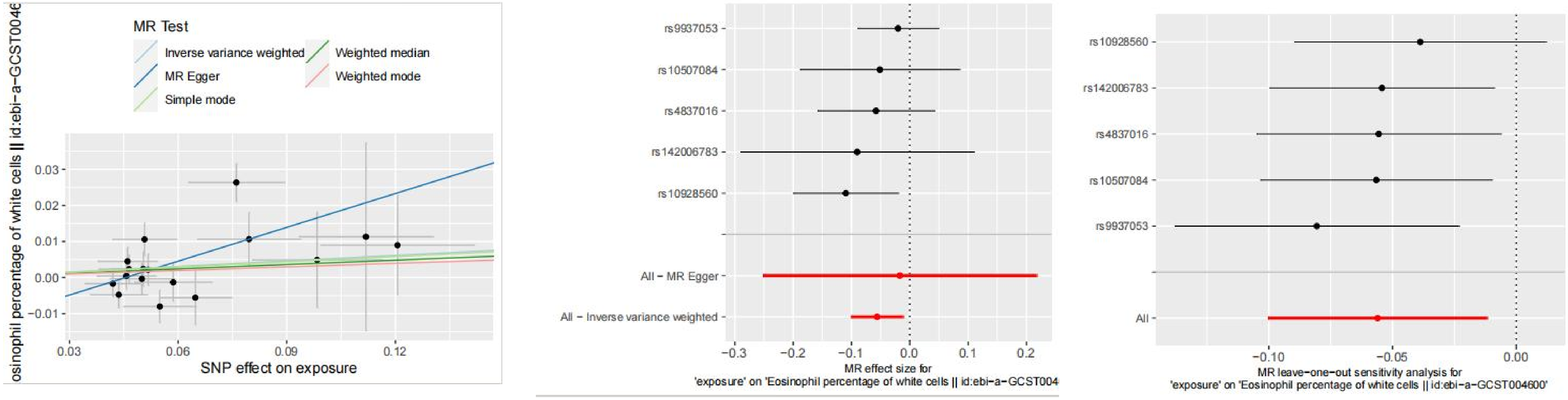

Scatter plot | Forest plot | Leave-one-out analysis

Figure S6. Genetic predicted OSA was causally associated with the decreased percentage of eosinophil on white cells.

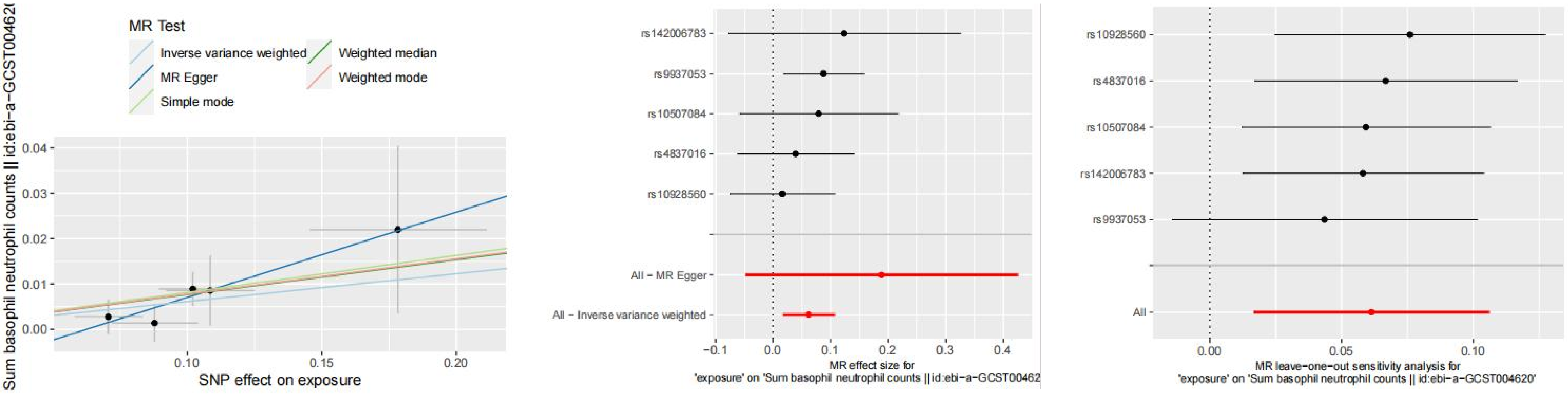

Scatter plot

Forest plot

Leave-one-out analysis

Figure S7. Genetic predicted OSA was causally associated with increased sum counts of basophil and neutrophil.

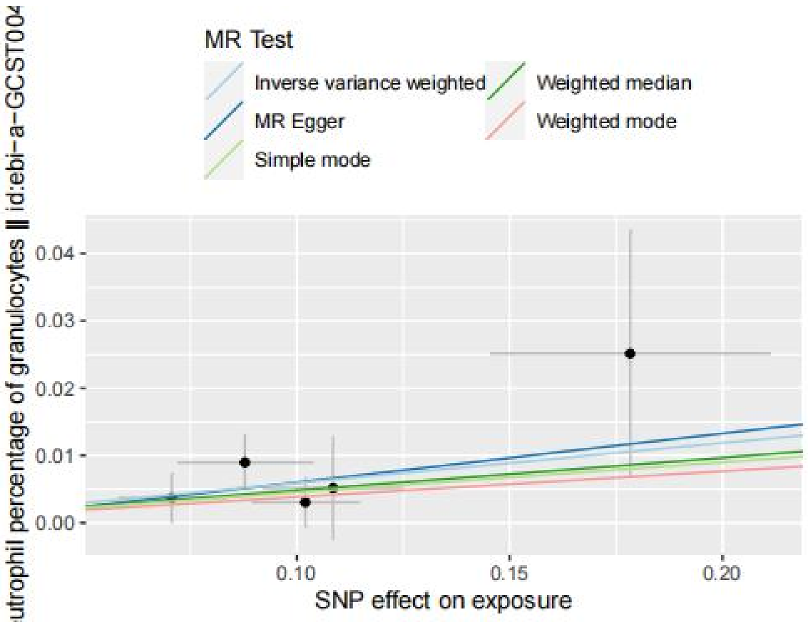

Scatter plot

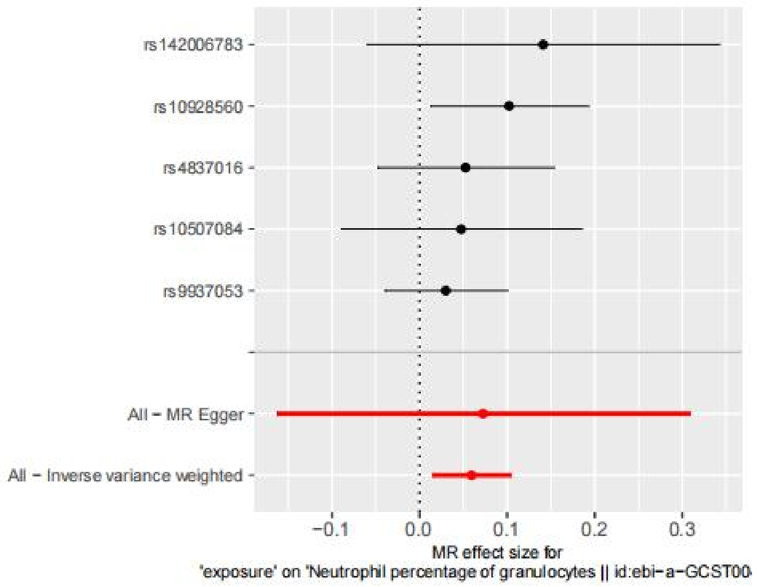

Forest plot

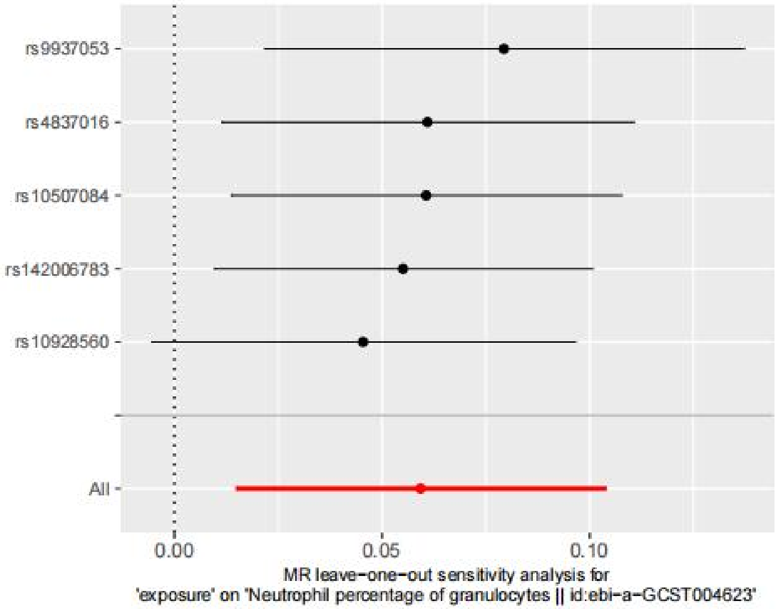

Leave-one-out analyses

Figure S8. Genetic predicted OSA was causally associated with increased neutrophil percentage of granulocytes.

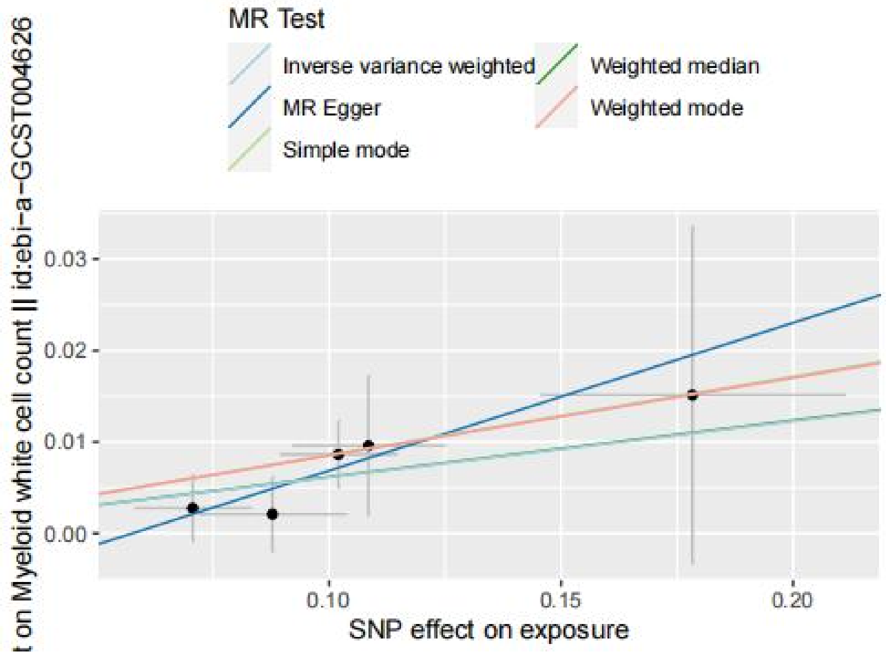

Scatter plot

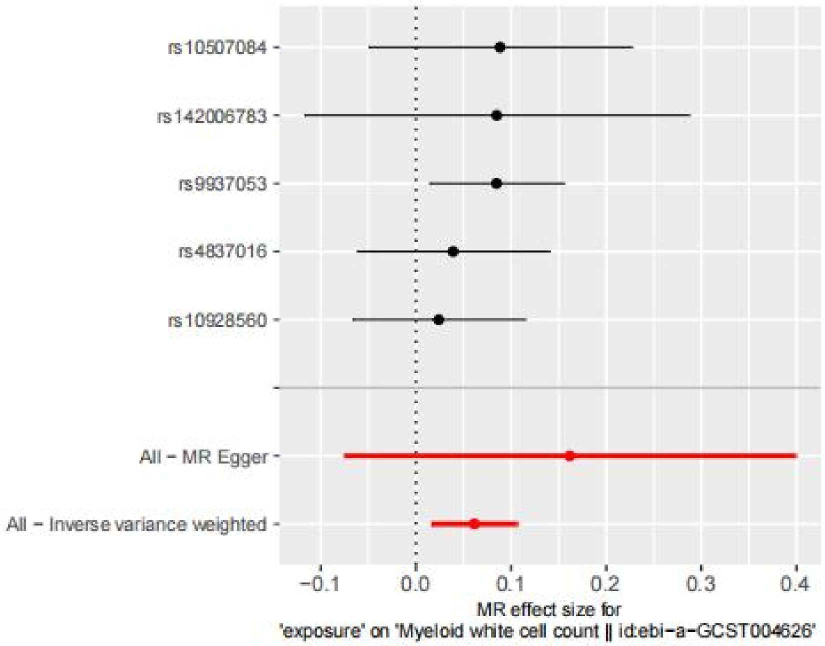

Forest plot

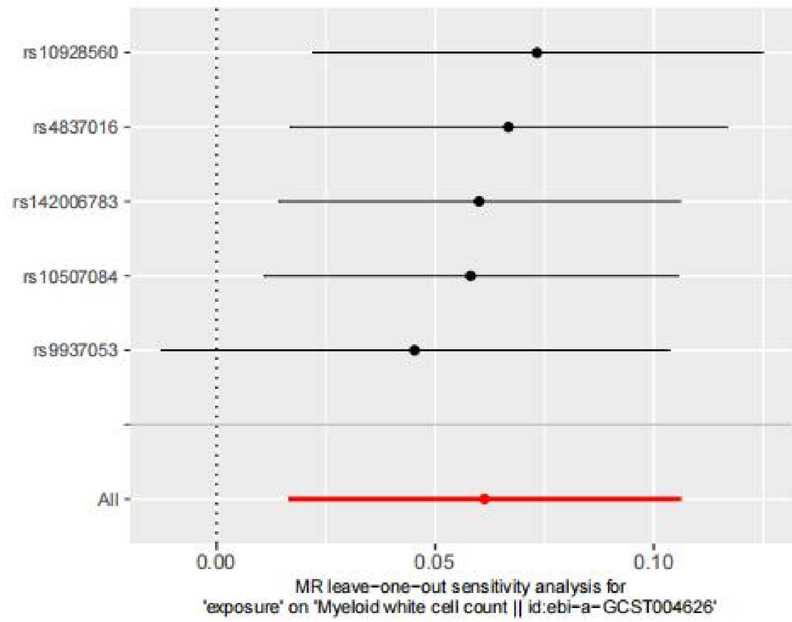

Leave-one-out analyses

Figure S9. Genetic predicted OSA was causally associated with increased myeloid white cell count.

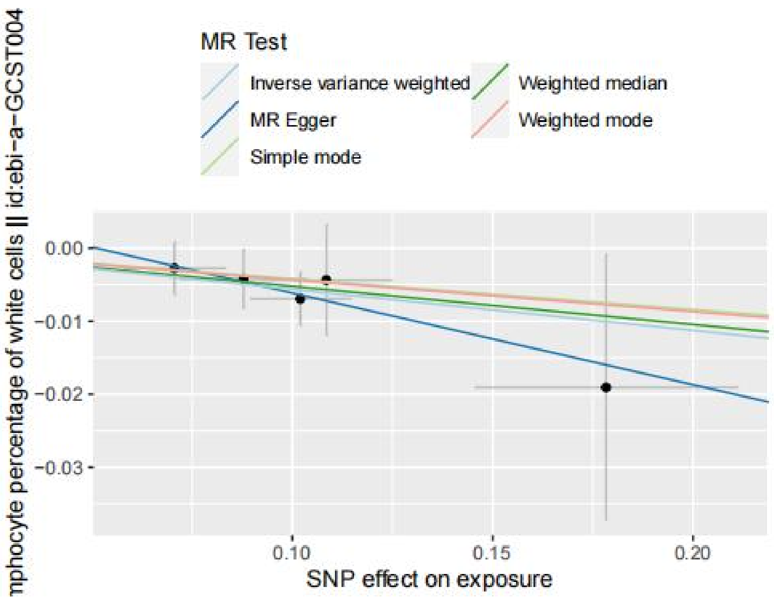

Scatter plot

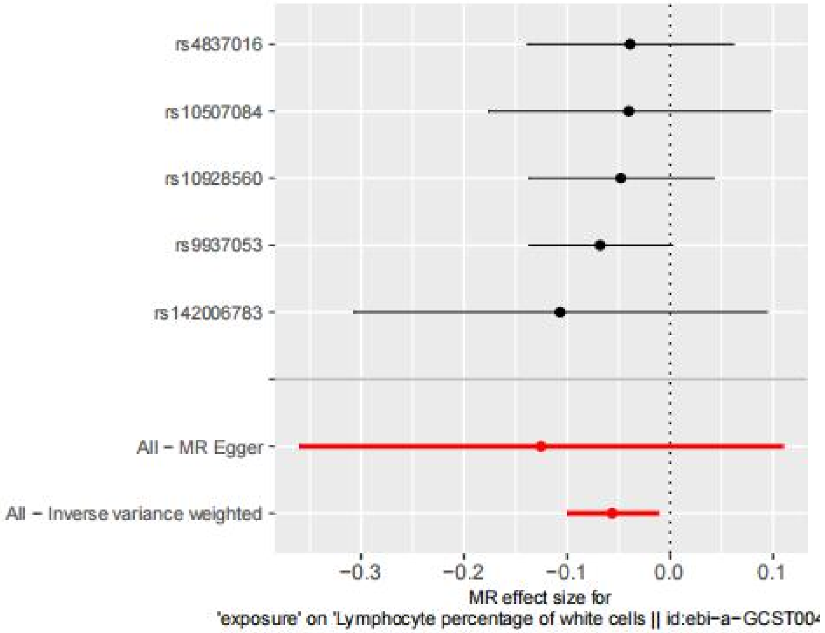

Forest plot

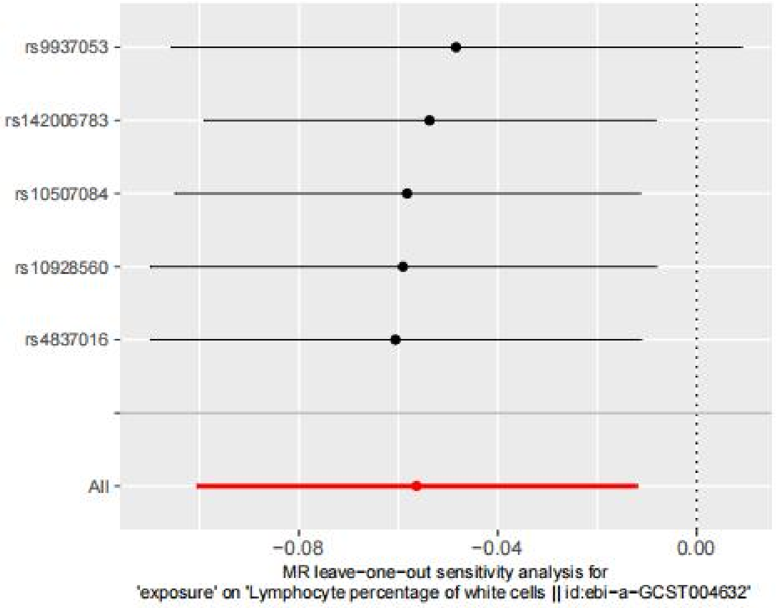

Leave-one-out analyses

Figure S10. Genetic predicted OSA was causally associated with decreased lymphocyte percentage of white cells.

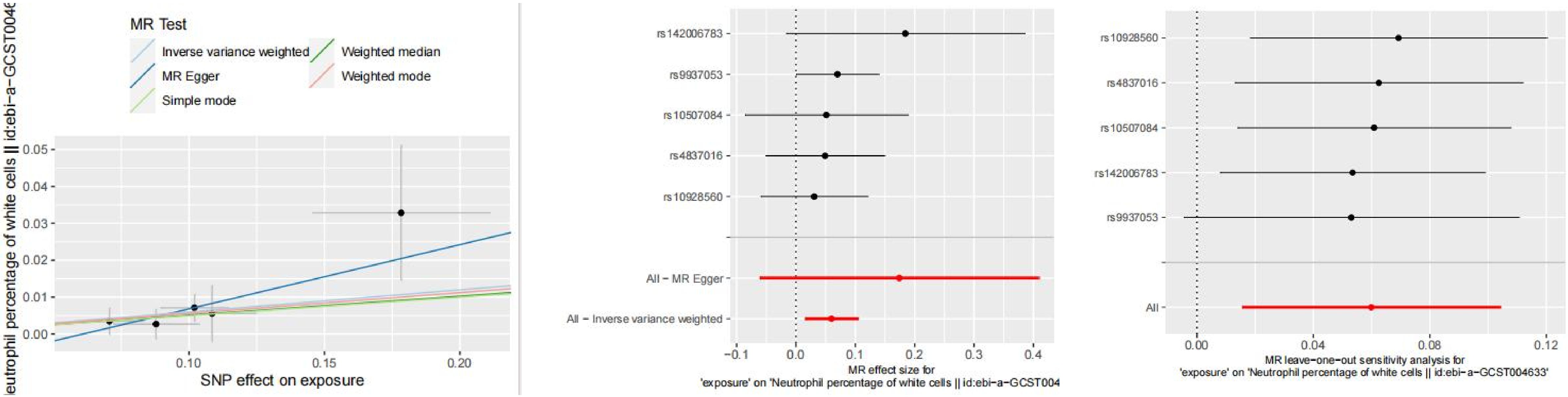

Scatter plot Forest plot Leave-one-out analyses

Figure S11. Genetic predicted OSA was causally associated with increased neutrophil percentage of white cells.

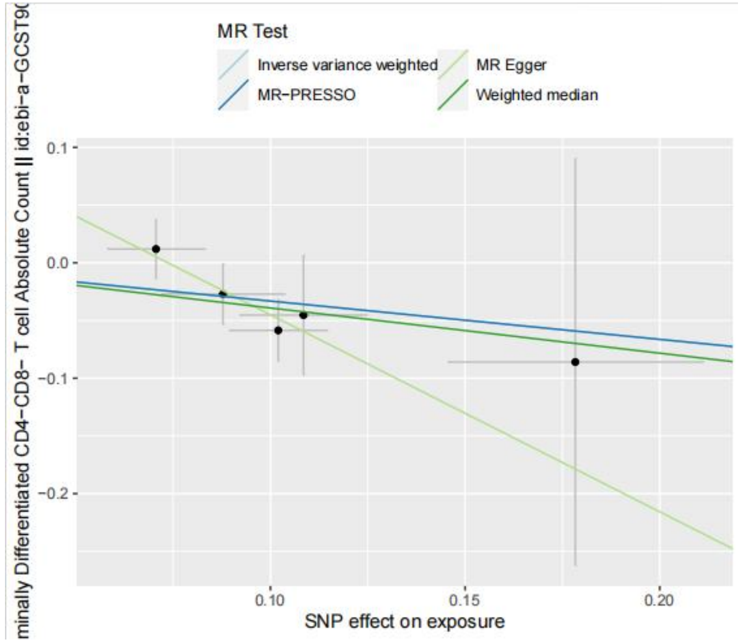

Scatter plot

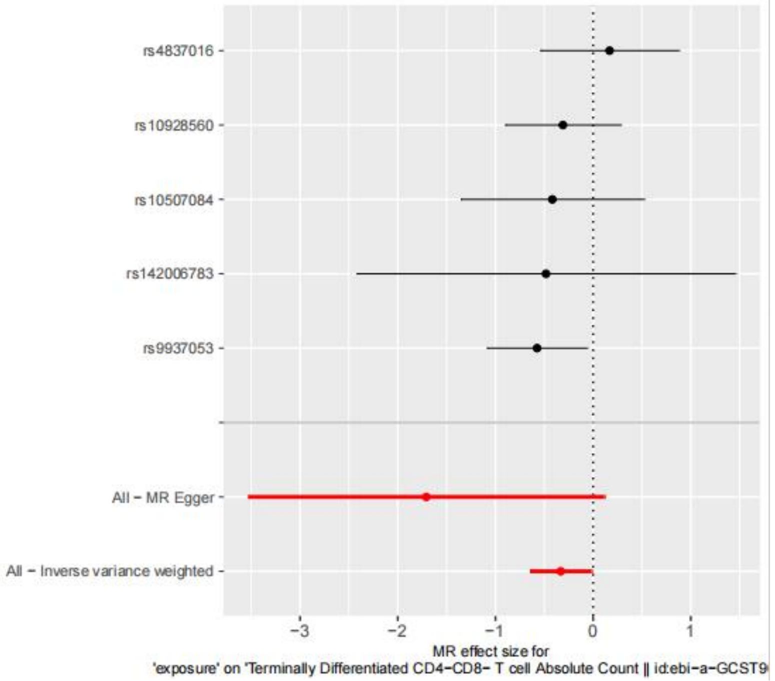

Forest plot

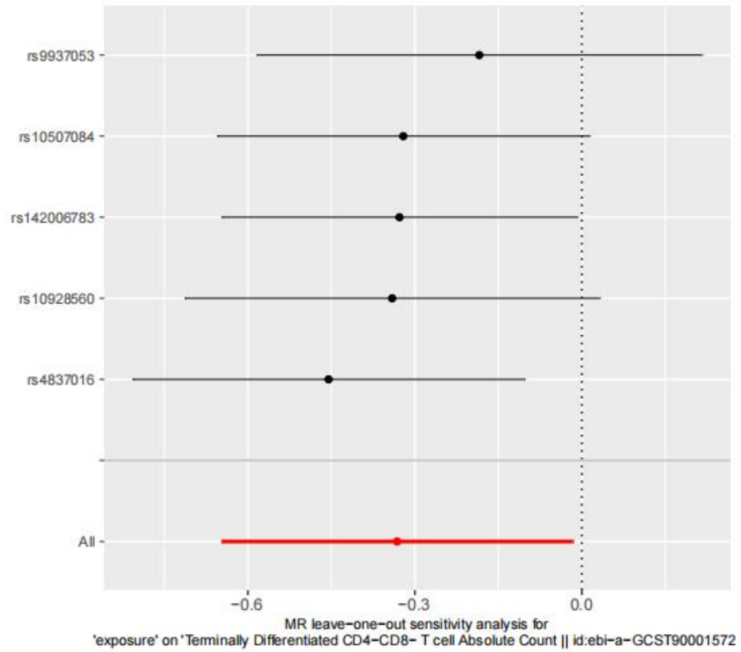

Leave-one-out analyses

Figure S12. Genetic predicted OSA was causally associated with decreased terminally differentiated CD4-CD8- T cell absolute count.

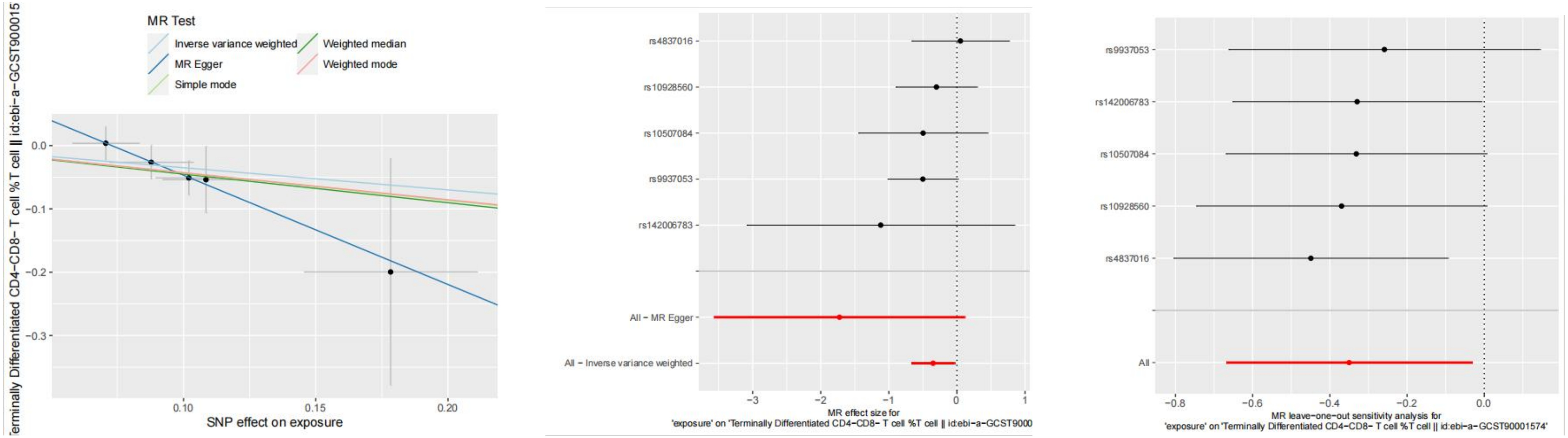

Scatter plot　　Forest plot　　Leave-one-out analyses

Figure S13. Genetic predicted OSA was causally associated with decreased percentage of terminally differentiated CD4-CD8- T cell on T cell.

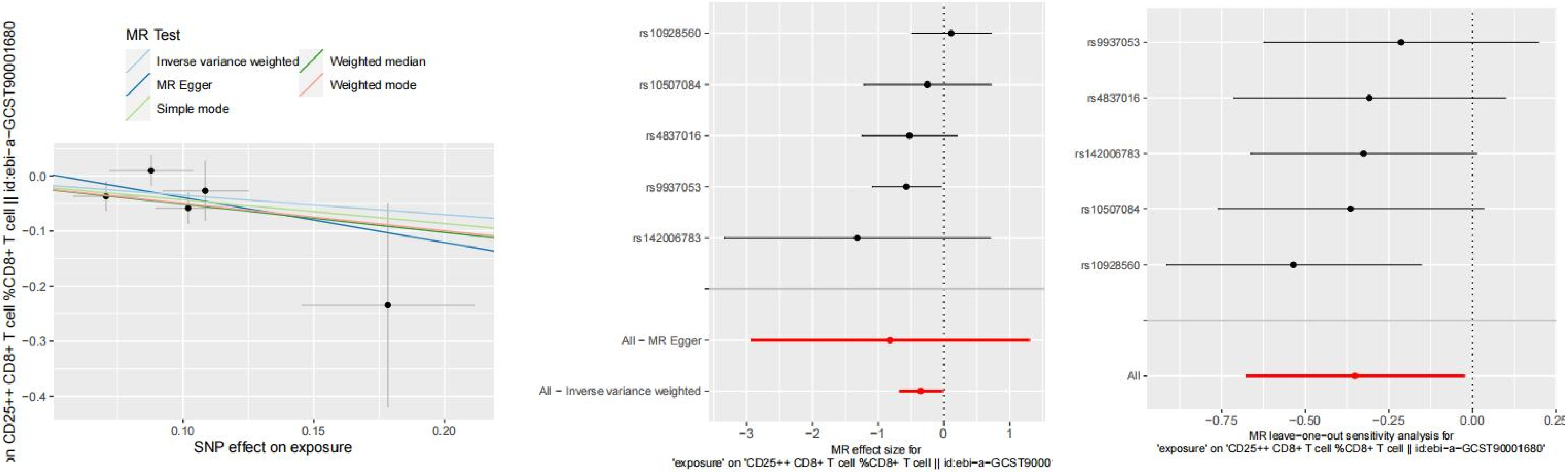

Scatter plot

Forest plot

Leave-one-out analyses

Figure S14. Genetic predicted OSA was causally associated with decreased percentage of CD25++ CD8+ T cell on CD8+ T cell.

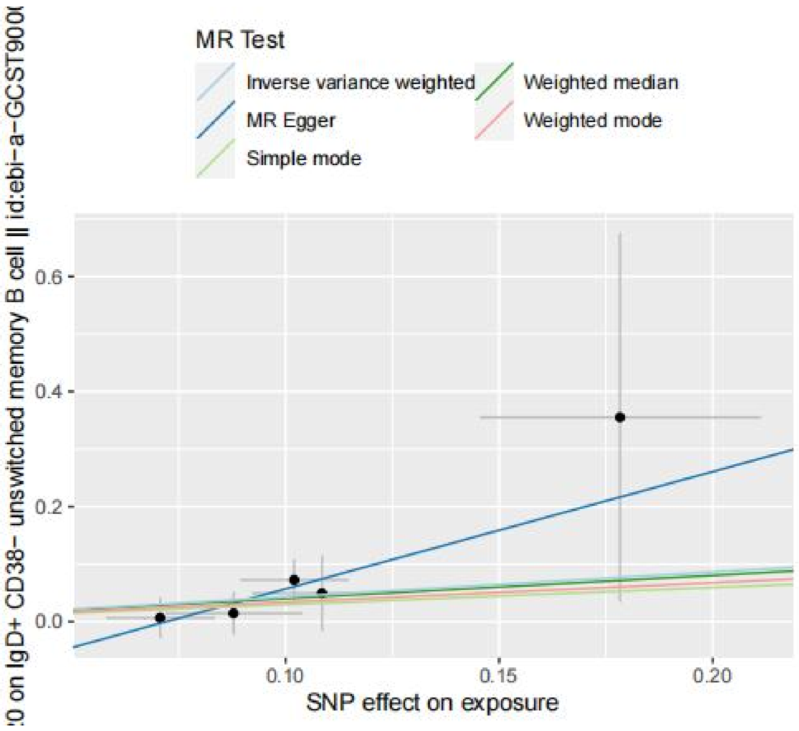

Scatter plot

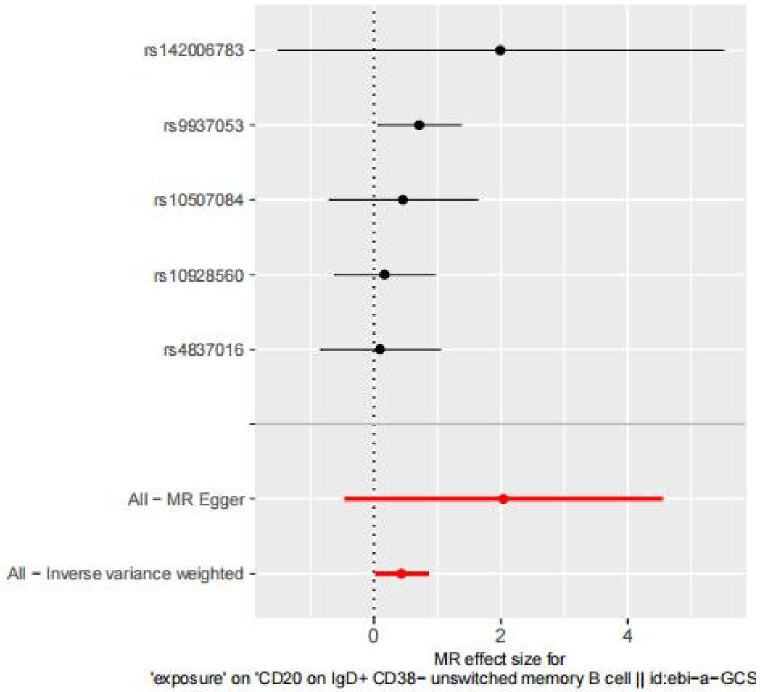

Forest plot

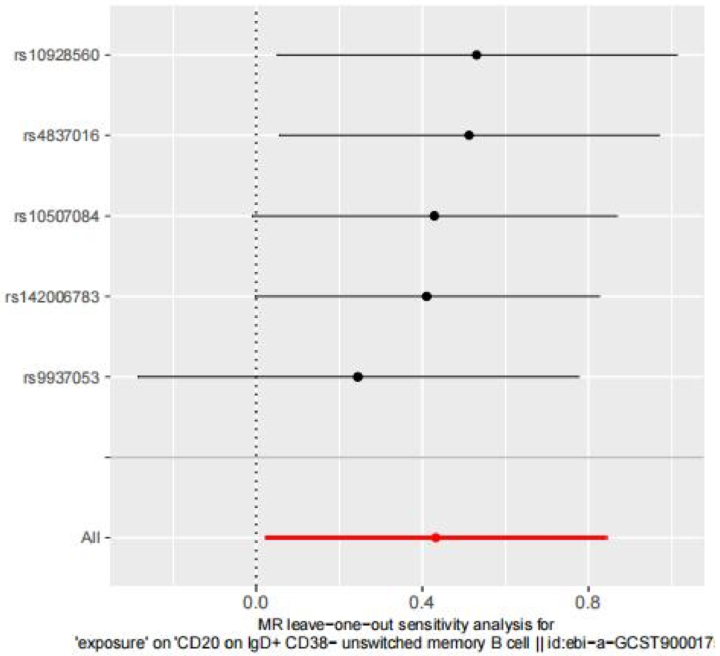

Leave-one-out analyses

Figure S15. Genetic predicted OSA was causally associated with increased percentage of CD20 on IgD+ CD38- unswitched memory B cell.

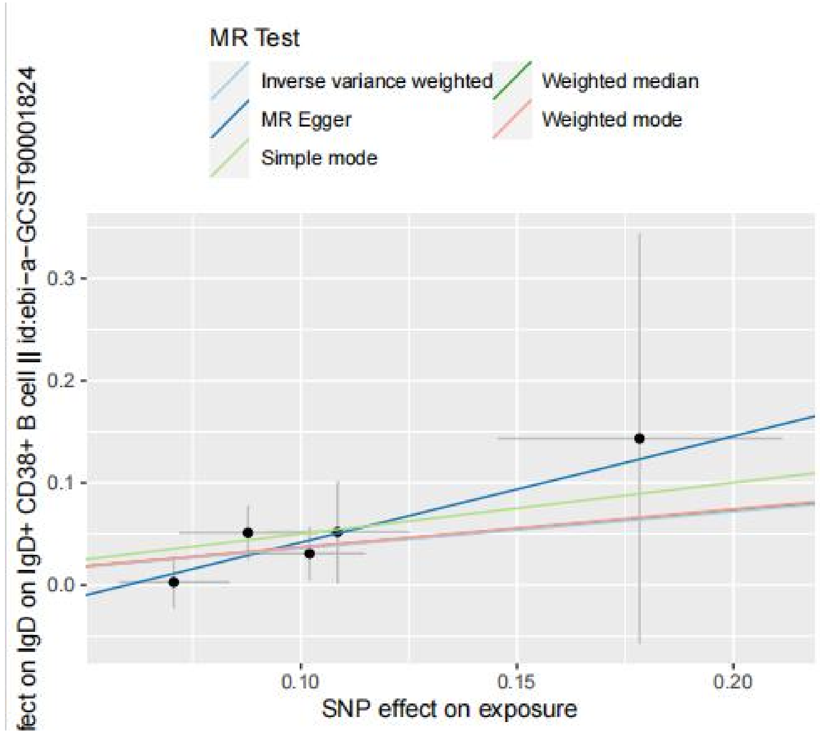

Scatter plot

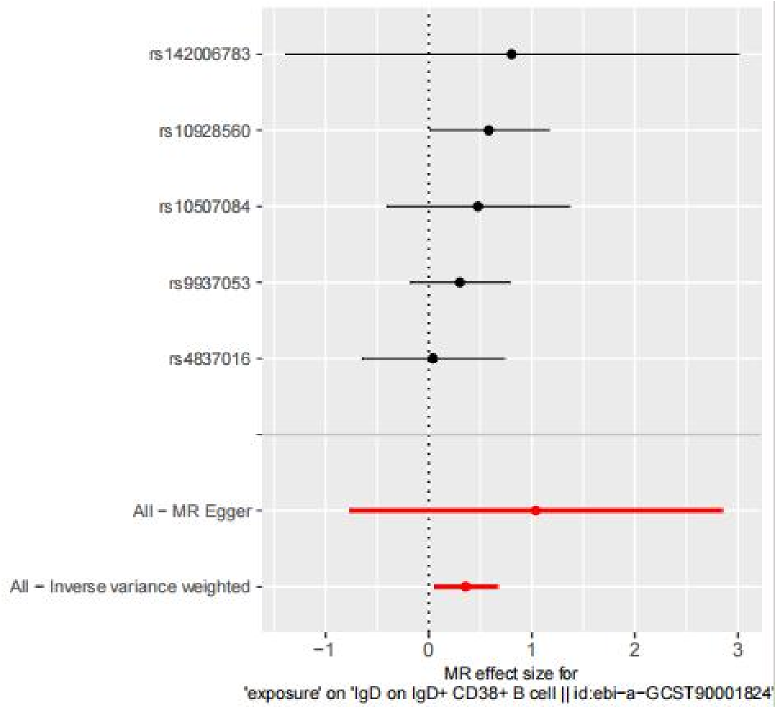

Forest plot

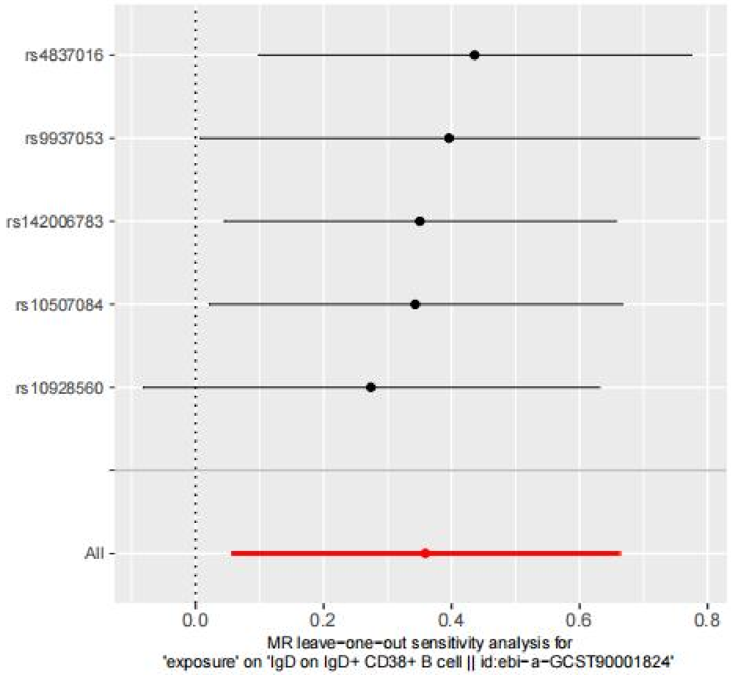

Leave-one-out analyses

Figure S16. Genetic predicted OSA was causally associated with increased percentage of IgD on IgD+ CD38+ B cell.

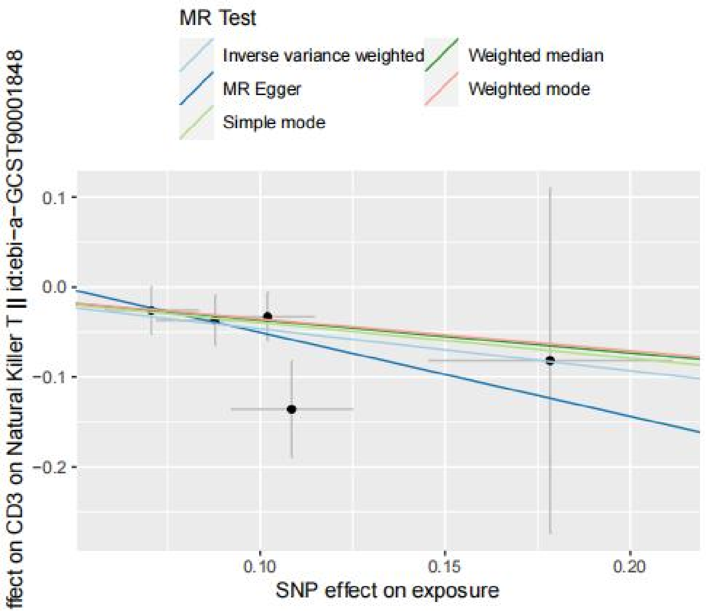

Scatter plot

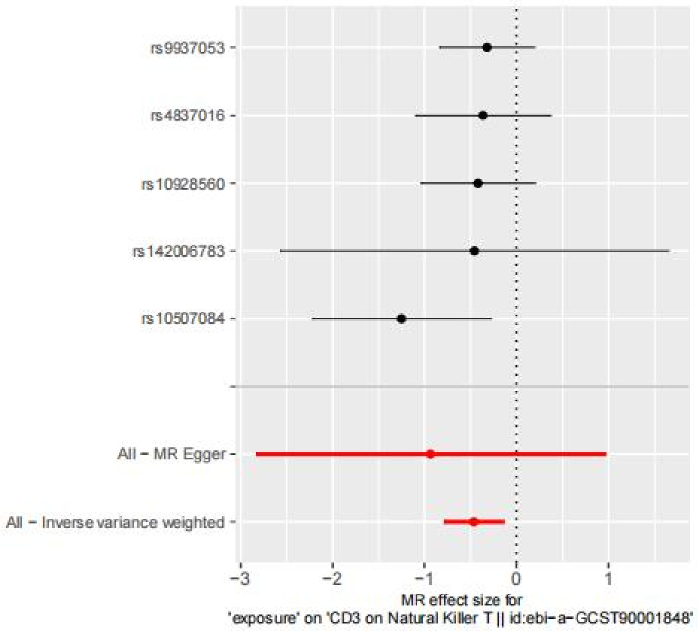

Forest plot

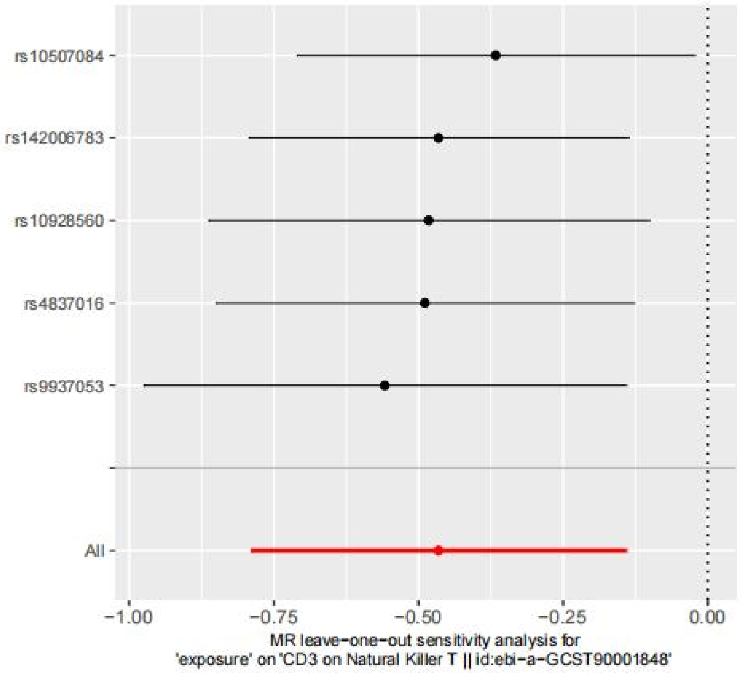

Leave-one-out analyses

Figure S17. Genetic predicted OSA was causally associated with decreased CD3 on Natural Killer T cell.

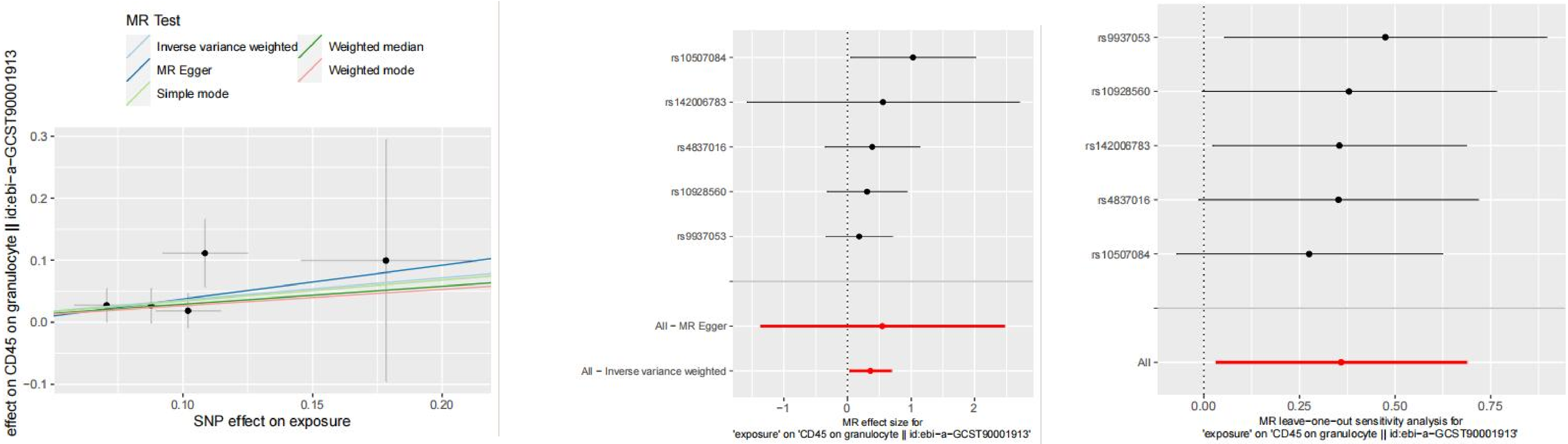

Scatter plot　　Forest plot　　Leave-one-out analysis

Figure S18. Genetic predicted OSA was causally associated with increased CD45 on granulocyte.

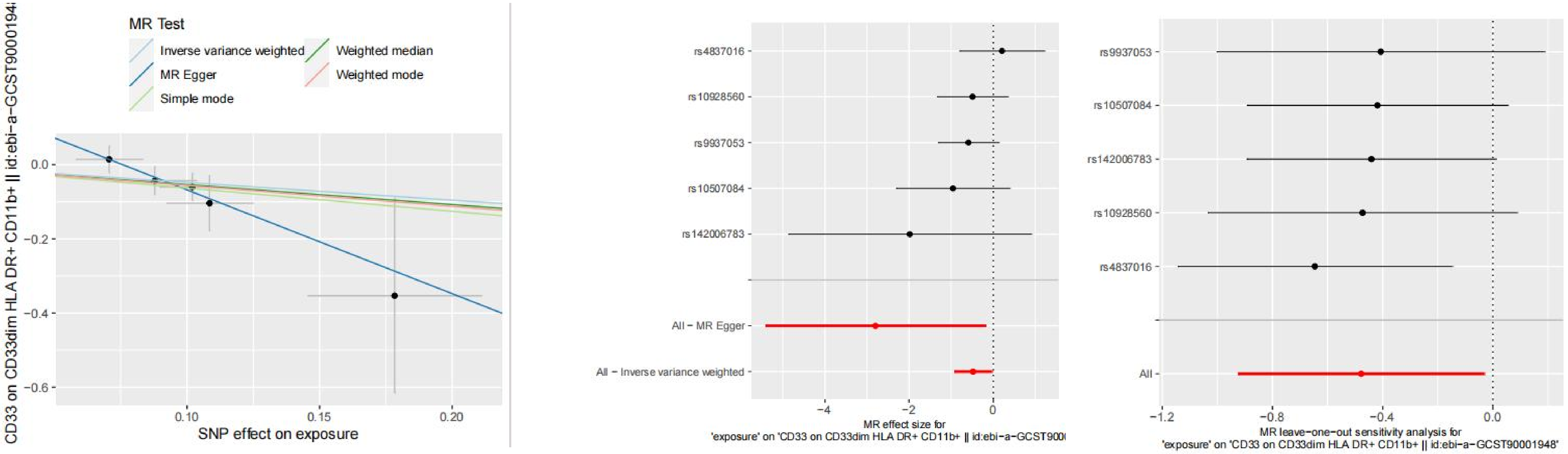

Scatter plot

Forest plot

Leave-one-out analyses

Figure S19. Genetic predicted OSA was causally associated with decreased the percentage of CD33 on CD33dim HLA DR+ CD11b+.

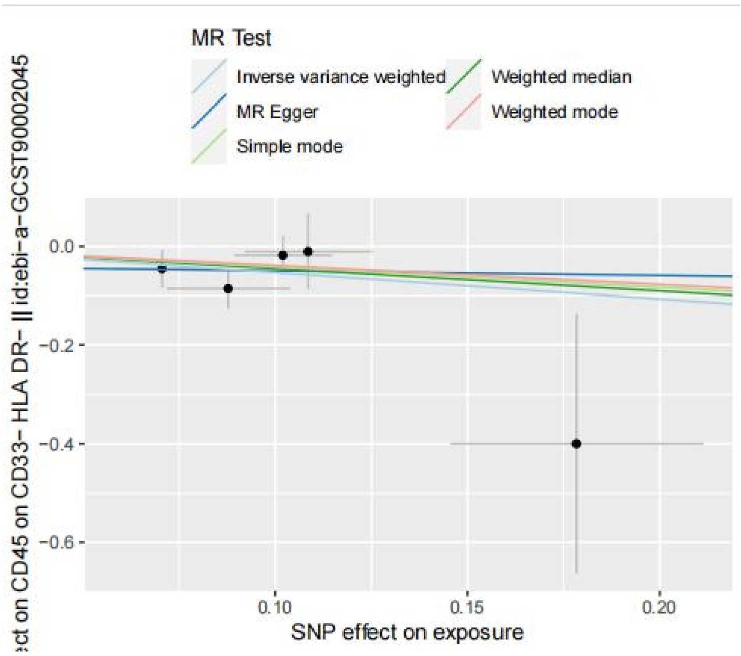

Scatter plot

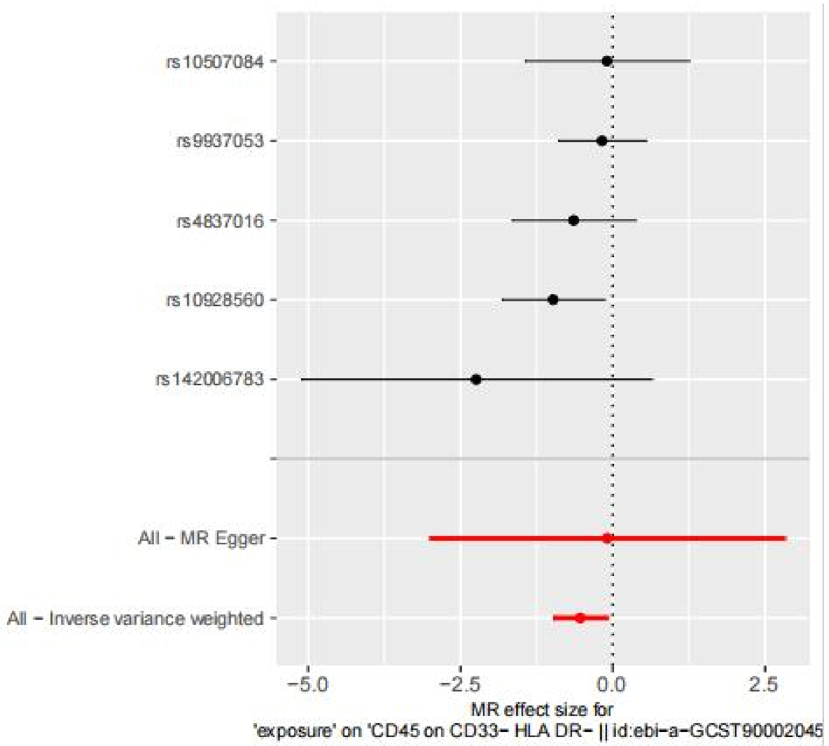

Forest plot

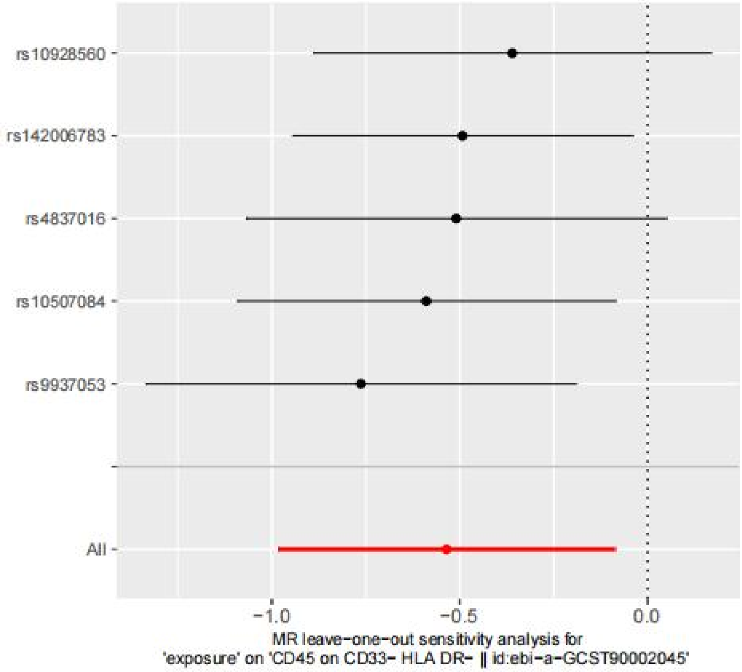

Leave-one-out analyses

Figure S20. Genetic predicted OSA was causally associated with decreased CD45 on CD33- HLA DR-.

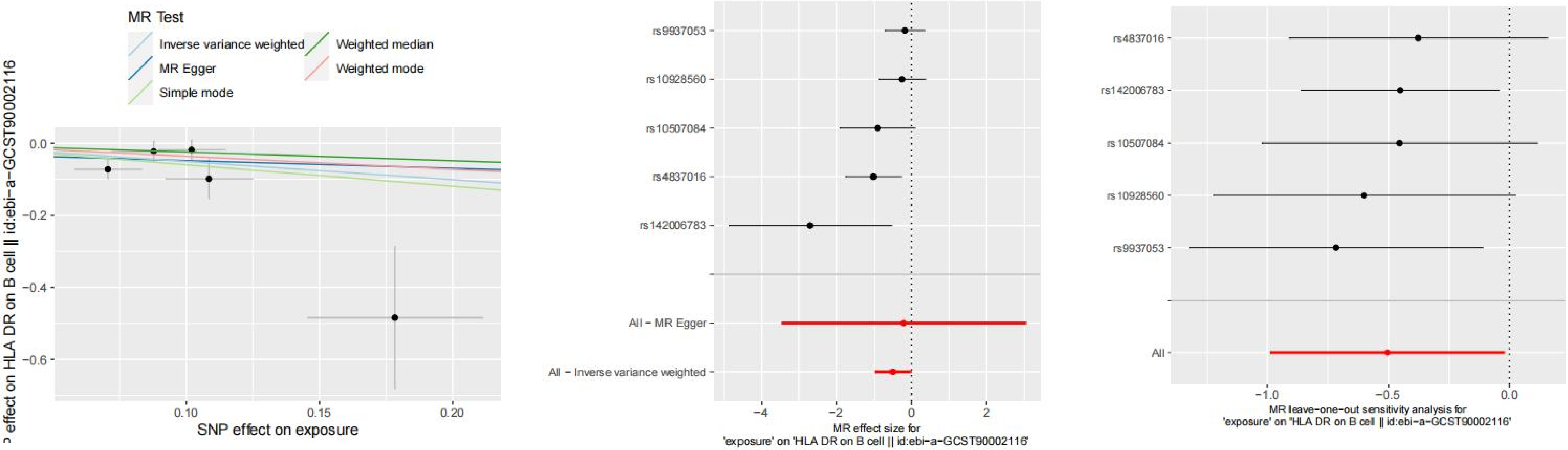

Scatter plot   Forest plot   Leave-one-out analyses

Figure S21. Genetic predicted OSA was causally associated with decreased HLA DR on B cell.
